# Simulation of *Pseudostellaria heterophylla* distribution in China: assessing habitat suitability and bioactive component abundance under future climate change scenariosplant components

**DOI:** 10.3389/fpls.2024.1498229

**Published:** 2024-12-04

**Authors:** Xu Li, Taosheng Wu, Chuangzhi Kang, Xiaobo Zhang, Jinqiang Zhang, Changgui Yang, Qingsong Yuan, Tao Zhou, Chenghong Xiao

**Affiliations:** ^1^ Resource Institute for Chinese and Ethnic Materia Medica, Guizhou University of Traditional Chinese Medicine, Guiyang, China; ^2^ National Resource Center for Chinese Materia Medica, China Academy of Chinese Medical Sciences, Beijing, China

**Keywords:** *Pseudostellaria heterophylla*, climate change, migration prediction, bioactive compound, species distribution

## Abstract

**Background:**

*Pseudostellaria heterophylla* is used in traditional Chinese medicine, so ensuring an adequate supply of plant material with high levels of bioactive components is important.

**Methods:**

Using an optimized maximum entropy niche model and assays of bioactive components from cultivation samples, this study started from the plant’s natural distribution area and estimated correlations of ecological factors with not only abundance of the plant but also abundance of polysaccharides and heterophyllin B. These correlations were combined with the spatial analysis function in ArcGIS to generate maps of the suitability of different habitats in China for cultivating *P. heterophylla* under current climate conditions and different models of climate change.

**Results:**

The following ecological factors emerged as particularly important for habitat suitability: precipitation of driest month and driest quarter, annual precipitation, annual mean temperature, temperature seasonality, and mean temperature of coldest quarter, contributing to a cumulative total of 87%. Under current climate conditions, optimum habitats of *P. heterophylla* were mainly distributed in the southwestern region (Guizhou) and eastern regions (Anhui, Zhejiang, Fujian, Jiangsu) of China, and only 0.197×10^6^ km^2^ of these areas were optimum habitat. In future climate change scenarios, the optimal habitat area of *P. heterophylla* exhibited an increase across different time periods under the SSP5-8.5 climate scenario. By the 2090s, distribution area of high heterophyllin B content under SSP5-8.5 climate scenarios will increase significantly, distribution area of high polysaccharide content had little change under all three climate scenarios (SSP 1-2.6, 2-4.5, 5-8.5). The center of mass of suitable habitat migrates southwestward under scenario SSP 1-2.6 and SSP 2-4.5, while it migrates northward under scenario SSP 5-8.5. Under the three climate scenarios, the center of mass of suitable habitat migrated consistently with that of high polysaccharide content but differed from that of high heterophyllin B content.

**Conclusion:**

These findings provide a crucial foundation for cultivating *P. heterophylla* with superior medicinal properties, developing adaptive management strategies to enhance conservation efforts, and ensuring sustainable utilization in the face of global climate change.

## 1 Introduction

Climate change is the dominant factor affecting the geographical distribution of vegetation (Kling et al., 2020; Huang et al., 2024) including its impact on species composition (Zhao et al., 2024) and habitat range (Zhang et al., 2024). The climatic environment directs effects not only on the growth, development, and distribution of plants but also on internal compositions (Neugart et al., 2018). The production of secondary metabolites in plants can be influenced by climatic changes, whether they are favorable or unfavorable (Sha et al., 2023; Cheng et al., 2024). Consequently, the biosynthesis of these compounds indirectly reflects the adaptability of plants to their environment (Mithofer and Boland, 2012). Given the speed and magnitude of global and regional climate change, it is crucial to clarify the factors that determine species distribution and their responses to such changes in order to accurately predict potential consequences for agriculture, forestry, and biodiversity conservation.

The geographical distribution and size of numerous medicinal plants have undergone substantial alterations as a consequence of global climate change (Groner et al., 2022; Shaban et al., 2023). Species distribution models (SDMs) (Courchesne et al., 2020) are mathematical models that utilize species distribution data and relevant environmental factors to predict the spatial distribution of suitable habitats, thereby elucidating the intricate interplay between species and their environment (Guisan and Thuiller, 2005; Waldock et al., 2021; Pratt et al., 2022). The ecological niche of medicinal plants has a significant impact on the accumulation of secondary metabolites (Applequist et al., 2020; Pant et al., 2021). Ecological models are currently being employed to predict the distribution of suitable habitats, cultivation areas, and environmental factors associated with the enhancement of active components in medicinal plants, making this a prominent research area. Niche modeling is a powerful technique for simulating species distribution (Rougier et al., 2015; Zhang et al., 2019). The application of this method enables accurate prediction of species’ current habitats and environmental requirements, thereby facilitating estimation of their potential distribution under various environmental conditions (Guisan and Zimmermann, 2000). This methodology facilitates the prediction of optimal habitats for species and evaluation of the relationships between species distribution and environmental variables (Jia et al., 2024). Such modeling has been used to explore the geographical distribution and future dynamics of autotrophic plants including *Notoginseng Radix* (Zhan et al., 2022)*, Zanthoxylum nitidum* (Yang et al., 2023)*, Cordyceps sinensis* (Wei et al., 2021)*, Codonopsis pilosula* (Wang et al., 2023)*, Coptis* (Li et al., 2020)*, Angelica sinensis* (Xu et al., 2020) and *Angelica dahurica* (Zhang et al., 2024).

The dried root of *Pseudostellaria heterophylla* (Miq.) Pax ex Pax et Hoffm., known as Pseudostellariae Radix in traditional Chinese medicine. It is commonly used for the treatment of fatigue, spleen asthenia, anorexia, asthenia after severe illness, and cough due to lung dryness (Commission, 2020). Recent pharmacological research has revealed that *P. heterophylla* possesses anti-diabetic properties (Chen et al., 2016) immune-enhancing effects (Yang Q. et al., 2020) and the ability to alleviate spleen deficiency (Xiao et al., 2022), due to its composition rich in various active compounds including pseudostellarin, polysaccharides, amino acids, saponins, and sapogenins (Hu D. J. et al., 2019). The wild *P. heterophylla* thrives in shady, warm, and humid forest habitats at elevations ranging from 800 to 2700 m above sea level across various provinces in China, including Liaoning, Inner Mongolia, Hebei, Shaanxi, Shandong, Jiangsu, Anhui, Zhejiang, Jiangxi, Henan and Hubei (Kang et al., 2016). Although wild *P. heterophylla* is rated as not endangered, wild resources are limited and the demand for this medicinal material is rising annually, the government has established a large-scale cultivation areas for it in Jiangsu, Fujian, Guizhou and Anhui Province of China (Jiang and Zhou, 2016). Climate change, by altering temperature and precipitation patterns, threatens to alter the suitability of different habitats for cultivating plant (Shen and Wang, 2013; Du et al., 2021) highlighting the importance of understanding what ecological factors are most important for its growth (Wang et al., 2019a). Furthermore, variations in the ecological conditions across different production regions contribute to notable disparities in the accumulation of active components and their quality within *P. heterophylla* (Wu M. et al., 2018; Hu D. J. et al., 2019; Hu D. et al., 2019). The aforementioned characteristics suggest that the consideration of *P. heterophylla* medicinal quality is essential when modeling habitat suitability. Investigating the ecological adaptability of *P. heterophylla* by examining the interplay between environmental factors and secondary metabolism is advantageous for species conservation and sustainable development. The absence of any reports forecasting the alterations in suitable cultivation regions and high quality areas of *P. heterophylla* under future climate change conditions is evident.

In the present study, we postulated that climate factors may exert an influence on both the distribution and quality of *P. heterophylla*. The relationship between climate factors and the suitable distribution of *P. heterophylla*, as well as the content of polysaccharide and heterophyllin B. The primary objectives of this study are as follows: (1) screening optimization model to predict the suitable habitats, and identify the key ecological factors influencing the distribution of *P. heterophylla*; (2) develop correlation models between ecological factors and bioactive component; (3) based on the correlation models, predict the change trend of suitable distribution area and high quality distribution area of *P. heterophylla* with climate change. The obtained results can serve as a scientific foundation for future production and conservation strategies of *P. heterophylla*.

## 2 Materials and methods

More detailed methods are provided in **Supplementary Table S1**.

### 2.1 Sample and data collection

**Figure 1:**
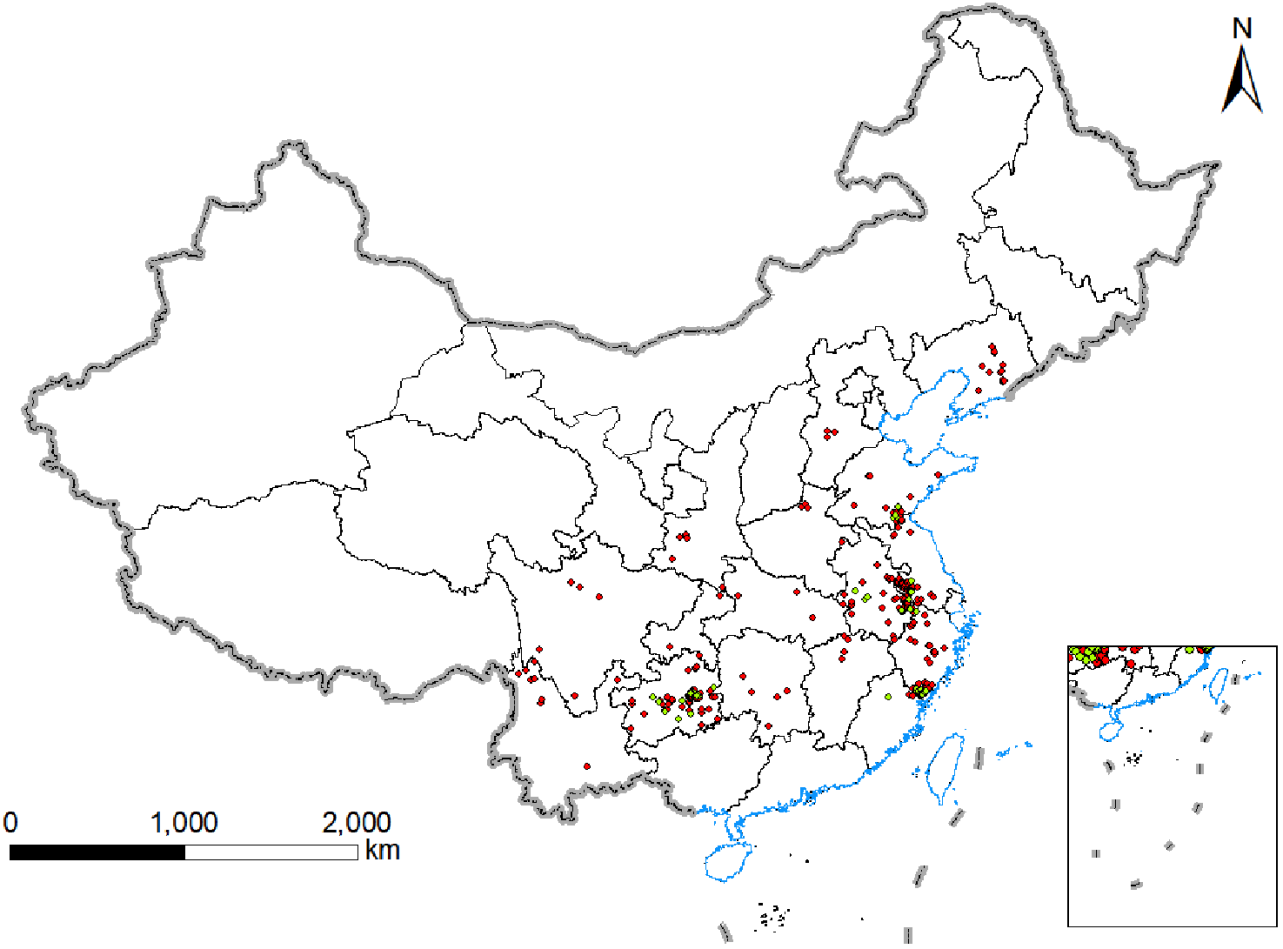
Geographical location of *P.heterophylla* samples in China.

We collected data on *P. heterophylla* distribution through the end of 2023 from the National Specimen Information Infrastructure (www.nsii.org.cn/2017/home.phpNSII), Chinese Virtual Herbarium (www.cvh.ac.cn), Plant Photo Bank of China (ppbc.iplant.cn), and literature indexed in the Chinese National Knowledge Infrastructure database. The “spThin” package in R (R Core Team, 2021) was used to delete data that were redundant or from locations within 10 km of a major sampling site in order to minimize variations due to sampling location (Aiello Lammens and Akçakaya, 2017). A total of 330 distribution records were retained in the final analysis (**Supplementary Table S2**; **Figure 1**).

These data from previous sampling were supplemented with triplicate sampling of *P. heterophylla* from 44 plots in Anhui, Shandong, Fujian, Jiangsu and Guizhou during the harvesting season between July and August (**Supplementary Table S3**; **Figure 1**).

### 2.2 Environmental variables

The environmental data used in this study came from the Worldclim version 2.1 (http://www.worldclim.org/) for 19 environmental variables, which were selected based on their relevance to plant survival and growth (Fick and Hijmans, 2017; Viljanen et al., 2024). Data were downloaded for the following periods: current period (1970–2000), future 2040s (averages for 2021-2040), 2060s (averages for 2041-2060), 2080s (averages for 2061-2080), and 2100s (averages for 2081-2100) (Debella-Gilo and Etzelmüller, 2009). In addition, The data of 55 ecological factors was acquired from the following sources: the soil data were obtained from the World Soil Database (http://www.crensed.ac.cn/), which was established by FAO and IIASA, terrain factor data were collected from the international scientific data service platform (http://datamirror.csdb.cn/), climatic data is sourced from the World Climate Database (http://www.worldclim.org/), which interpolates recorded data from various meteorological stations worldwide between 1950 and 2000. It comprises 43 climate type data (including 12-month rainfall and average gas temperature, as well as 19 comprehensive climate factors), 8 soil type data (such as soil pH, cation exchange capacity, sand content, clay content, soil subcategory, effective water content grade, soil texture classification, and organic carbon content), three topographic data (elevation, slope degree and slope direction), and vegetation types (**Supplementary Table S4**).

Predicted future climate data for China were downloaded from the Beijing Climate Center Climate System Model (BCC-CSM2-MR) from the sixth phase of the Coupled Model Intercomparison Project (Wu T. et al., 2018; Sang et al., 2020; Jin et al., 2021; Kumar and Sarthi, 2021; Wang et al., 2021). Data covering the same time periods were downloaded for the shared socioeconomic pathway (SSP) 1-2.6, corresponding to low compulsion; SSP2-4.5, corresponding to medium radiative forcing; and SSP5-8.5, corresponding to high compulsion. These SSPs were chosen because of their relatively high accuracy and resolution and because they take into account local development (He et al., 2017; Liu et al., 2021). To eliminate collinearity, we performed Pearson’s correlation analysis across environmental variables and Exclude high-correlation environmental variables (|r| ≥ 0.8), for factors with a correlation coefficient exceeding 0.8, only those with greater ecological significance were retained (**Supplementary Figures S1**, **S2**).

To ensure consistency with the data format requirements of the MaxEnt 3.4.1 model for environmental variables, resolution of all environmental variables was resampled to 2.5′(5 km×5 km) using ArcGIS 10.4 software (Esri.(2020).ArcGIS Desktop: Release 10.4. Redlands, CA: Environmental Systems Research Institute.).

### 2.3 Model optimization and evaluation

Using the Biomod2 package (v4.3.1, https://cran.r-project.org/bin/windows/base/). Nine algorithms were applied in this study: SRE (Surface Range Envelope), RF (Random Forest), FDA (Flexible Discriminant Analysis), CTA (Classification Tree Analysis), GAM (Generalized Additive Model), ANN (Artificial Neural Network), GLM (Generalized Linear Model), MARS (Multivariate Adaptive Regression Splines), MaxEnt (Maximum Entropy) (Zhao Y. et al., 2021). ENMeval optimized feature class setting and *β* regularization multiplier are used, and the other algorithms use Biomod2 default model setting. The Biomod2 is widely used to predict species distribution and test species distribution model (Huang D. et al., 2023; Huang L. et al., 2023; Suicmez and Avcı, 2023) From the distribution data points in the model, 75% were randomly selected to train the model, and the remaining 25% were used to evaluate model performance in 10 replications (Muscarella et al., 2015). Algorithm performance was assessed in terms of average AUC (the area under the subject operating characteristic curve) and TSS (true skill statistic values) of single and ensemble model (Warren and Seifert, 2011).

The ENMeval package in R software was used to optimize the selective model. The ‘regularization multiplier’ and ‘feature combination’ modules in the ENMeval package within R were used to reduce model complexity and improve model accuracy (Deka and Morshed, 2018). The value of the regularization multiplier ranged from 0.5 to 4, and it was increased in steps of 0.5 for a total of eight frequency doublings. Nine feature combinations were applied: “L”, “LQ”, “H”, “LQH”, “LQHP”, “LQHPT”, “QHP”, “QHPT”, and “HPT”, where L was linear; Q, quadratic; H, hinge; P, product; and T, threshold. Altogether 72 parameter combinations were tested.

### 2.4 Spatial analysis of habitat suitability for *P. heterophylla*

The spatial distribution of suitable habitats for *P. heterophylla* was output as raster data from ArcGIS 10.4.1 (Esri.(2020).ArcGIS Desktop: Release 10.4.Redlands,CA: Environmental Systems Research Institute.) with attributes of the setting layer defined using the “reclassify” tool according to the natural breakpoint method (Guiquan et al., 2023). Habitats were classified as unsuitable if their fitness value was 0-0.1; secondarily suitable, 0.2-0.25; suitable, 0.26-0.5; or optimal, 0.6-1.

In this study, the SDMTool package in R software was used to calculate the change trends in the suitable region and the geometric center positions of the suitable regions in the modern era and the future. We considered the suitable *P. heterophylla* habitat as a whole and reduced it to a vector particle and used the change of the position of the centroid to reflect the size and direction of the species’ suitable habitat. Finally, The center of mass was tracked using different SDMs to examine the centroid of *P. heterophylla* during various periods and under diverse climatic conditions, in order to evaluate the migration distance of the suitable *P. heterophylla* zone in latitude and longitude coordinates (Brown et al., 2017).

### 2.5 Analysis of multivariate environmental similarity surface and most dissimilar variable

A multivariate environmental similarity surface was generated for the current distribution of suitable habitats as well as for future distributions predicted under different climate change scenarios in order to explore the degree of climatic anomaly in current and future distributions. The surface was generated relative to a reference layer based on the environmental variables in the current distribution area of *P. heterophylla*. The surface indicates the degree of similarity between a set of predictive variables (V1, V2, Vi,…) and a set of reference points. In the reference layer, mini and maxi are the minimum and maximum values of the environmental variable Vi; pi, the value of the environmental variable Vi at a certain point P during the given period; and fi, the percentage of points where Vi< pi (Muscatello et al., 2021). When fi = 0, the surface is 100 (p - mini)/(maxi - mini); when 0< fi ≤ 50, the surface is 2fi; when 50< fi< 100, the surface is 2 (100 - fi); and when fi = 100, the surface is 100 (maxi-100)/(maxi-mini). If the surface is negative, it indicates that the value of at least one variable falls outside the environmental range of the reference point during the given period, which is called the climate anomaly point. When the value of the point is 100, it indicates that the climate environment is completely consistent with the reference layer, and the climate is normal.

Environmental factors that most heavily influenced modeled changes in distribution of suitable habitats were identified based on the “most dissimilar variable” (Zhao G. et al., 2021) defined as the minimum similarity value for the surface across different environmental variables at point P. The most dissimilar variable is also the variable showing the greatest anomaly and therefore a variable likely to strongly influence the geographic migration of species. This variable was determined using the “novel density” (Yao et al., 2016) tool in the maximum entropy model.

### 2.6 Determination of polysaccharide and heterophyllin B in Pseudostellariae Radix

A total of 44 samples of *P. heterophylla* from the sites indicated in (**Figure 1**) were assayed for heterophyllin B according to the Chinese Pharmacopoeia.

They were assayed for polysaccharide content as follows. An aliquot of Pseudostellariae Radix powder (approx. 0.1 g) was accurately weighed, added to a 100 mL round-bottom flask, dissolved in 70 mL of 80% ethanol, heated in a water bath, refluxed for 30 min, and filtered while still hot. The residue and filter paper were placed in a clean flask, 80 mL of water was added, the suspension was soaked for 1 h in a water bath at 90 °C, it was filtered while it was still hot, the residue was washed three times with hot water (5 mL each time), and the washes were pooled together with the filtrate. The solution was allowed to cool and transferred to 100 mL volumetric flask. An aliquot (2.0 mL) was transferred into a 25 mL test tube and mixed well with 1 mL of 4% phenol solution, immediately after which 5 mL of concentrated sulfuric acid was added dropwise and the solution was shaken well each time. The solution was allowed to stand at room temperature.

To prepare a reference solution for polysaccharides, glucose was dried to constant weight (15.07 mg) in a 25 mL volumetric flask, and water was added to yield a final glucose concentration of 602.8 µg·mL^-1^. A series of calibration solutions were prepared by transferring 0.5, 0.8, 1.0, 1.3, 1.5 and 1.8 mL of the reference solution into 25 mL measuring bottles, diluted with water and shaken well.

Absorbance of the reference solutions and Pseudostellariae Radix samples was measured at 489 nm. The calibration curve showed excellent linearity over the glucose concentration range from 3.0140 to 10.8505 µg·mL^-1^ [absorbance = (0.0548 glucose concentration) + 0.0042, r = 0.9996]. Polysaccharide content was defined as


Polysaccharide content=[(c×d×f)/w]×100%

where c is the concentration of glucose (µg·mL^-1^) among the polysaccharides in the sample, d is the polysaccharide dilution factor, f is the polysaccharide conversion factor (2.38), and w is the sample weight (g).

Associations of ecological factors with content of polysaccharides and heterophyllin B in Pseudostellariae Radix were explored using stepwise regression in SPSS 26.0(IBM, Chicago, IL, USA).

## 3 Results

### 3.1 Identification and optimization of the most appropriate niche model

**Figure 2:**
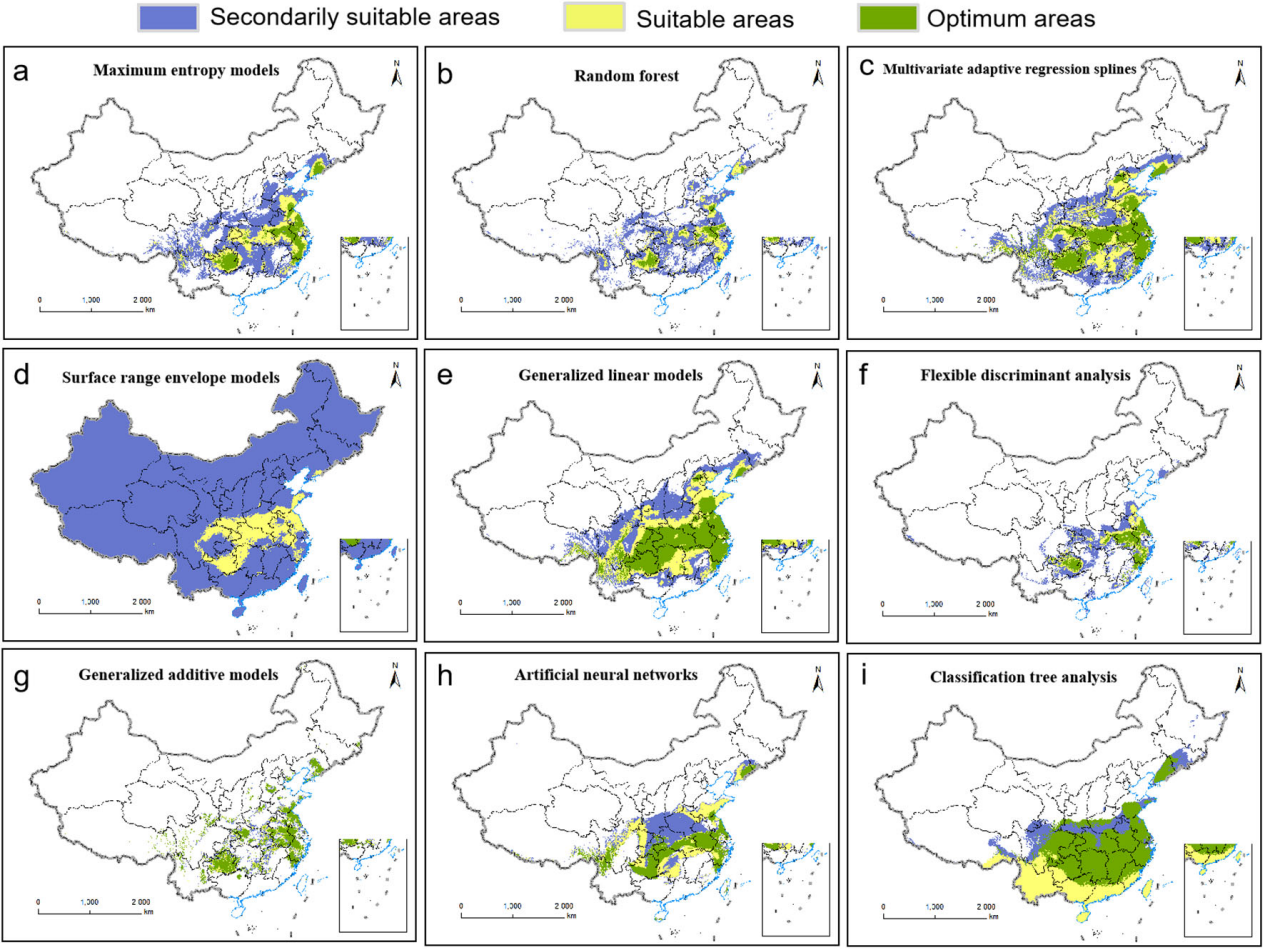
Current distributions of *P.heterophylla* in China as predicted by nine niche models: **(A)** Maximum Entropy. **(B)** Random Forest. **(C)** Multivariate Adaptive Regression Splines. **(D)** Surface Range Envelope. **(E)** Generalized Linear Model. **(F)** Flexible Discriminant Analysis. **(G)** Generalized Additive Model. **(H)** Artificial Neural Network. **(I)** Classification Tree Analysis.

**Figure 3:**
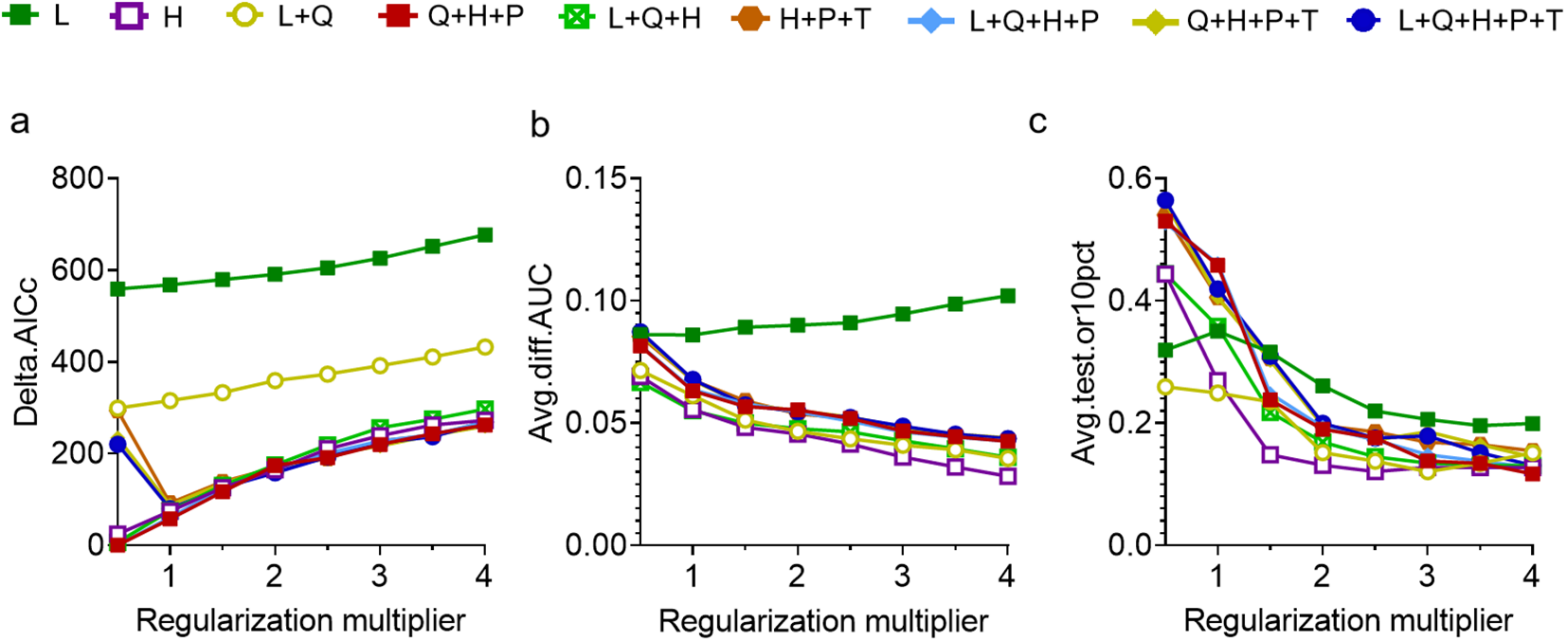
Optimization of the maximum entropy model based on **(A)** AICc. **(B)** AUC difference. **(C)** 10% training omission rate. Feature combinations are abbreviated as follows: H, hinge; L, linear; P, product; Q, quadratic; T, threshold.

Based on 330 occurrence records and 19 environmental variables, the AUC and TSS values of nine species distribution niche models (SRE, RF, FDA, CTA, GAM, ANN, GLM, MARS, MaxEnt) were compared using the Biomod2 platform. The results are presented in **Table 1**. Among the tested models, MaxEnt exhibited higher AUC values (0.969), followed by RF (0.956) and MARS (0.950). Similarly, MaxEnt demonstrated higher TSS values of 0.845 along with ANN (0.808) and RF (0.786). Notably, MaxEnt outperformed all other models with its largest AUC and TSS values indicating superior prediction accuracy. Furthermore, the current distribution predicted by this model showed the most accurate fit to the actual plant distribution (**Figure 2**). Consequently, we selected the MaxEnt for subsequent analysis. This model was optimized by testing eight values of the regularization multiplier from 0.5 to 4 as well as nine combinations of parameters (L, LQ, H, LQH, LQHP, LQHPT, QHP, QHPT, HPT), giving a total of 72 combinations of multipliers and parameters. Applying a regularization multiplier of 1 and combination of features LQH gave an AICc = 0 as well as an AUC that was 76.38% lower and a 10% training omission rate that was 59.55% lower than with default parameters (**Figure 3**). This optimized model was used in all subsequent analyses.

**Table 1:** Comparison of nine niche models for predicting current *P. heterophylla* distribution in China.

Model	Area under the curve	True skill statistic
Maximum Entropy	0.969 ± 0.004	0.845 ± 0.021
Random Forest	0.956 ± 0.020	0.786 ± 0.028
Multivariate Adaptive Regression Splines	0.950 ± 0.007	0.774 ± 0.027
Surface Range Envelope	0.836 ± 0.022	0.672 ± 0.045
Generalized Linear Model	0.914 ± 0.004	0.757 ± 0.021
Flexible Discriminant Analysis	0.934 ± 0.018	0.747 ± 0.019
Generalized Additive Model	0.907 ± 0.022	0.764 ± 0.036
Artificial Neural Networks	0.945 ± 0.022	0.808 ± 0.026
Classification Tree Analysis	0.916 ± 0.025	0.734 ± 0.030

Values are mean ± SD of 10 runs.

### 3.2 Identification of the most important environmental determinants of *P. heterophylla* distribution

We utilized 55 ecological factors to screen for the primary determinants influencing the distribution of *P. heterophylla* (**Supplementary Table S5**), and ascertained that all of them were climate-related variables. Consequently, in this investigation, we narrowed down our focus to 19 climatic ecological factors when screening for the principal determinants impacting the distribution of *P. heterophylla*.

The jackknife test was employed to evaluate the contribution of each ecological factor in the MaxEnt to the predicted distribution of *P. heterophylla*. Environmental factors with a contribution exceeding 4% were identified as primary factors. Six main factors were determined, collectively accounting for 87% of the distribution (**Table 2**): Precipitation of driest month accounted for 31%; annual precipitation accounted for 18%; precipitation of driest quarter accounted for 15.8%; mean annual temperature accounted for 11.7%; temperature seasonality (standard deviation *100) accounted for 5.5%; mean temperature of coldest quarter accounted for 5%.

**Table 2:** Contribution of individual environmental variables to MaxEnt prediction of the current distribution of *P. heterophylla* in China.

Variables ID	Description	Contribution (%)	Permutation importance(%)	Units
Bio14	Precipitation of driest month	31	2.8	mm
Bio12	Annual precipitation	18	37.1	mm
Bio17	Precipitation of driest quarter	15.8	1.3	mm
Bio1	Annual mean temperature	11.7	6.9	°C
Bio4	Temperature seasonality (standard deviation*100)	5.5	10.6	—
Bio11	Mean temperature of coldest quarter	5	15.7	°C

**Figure 4:**
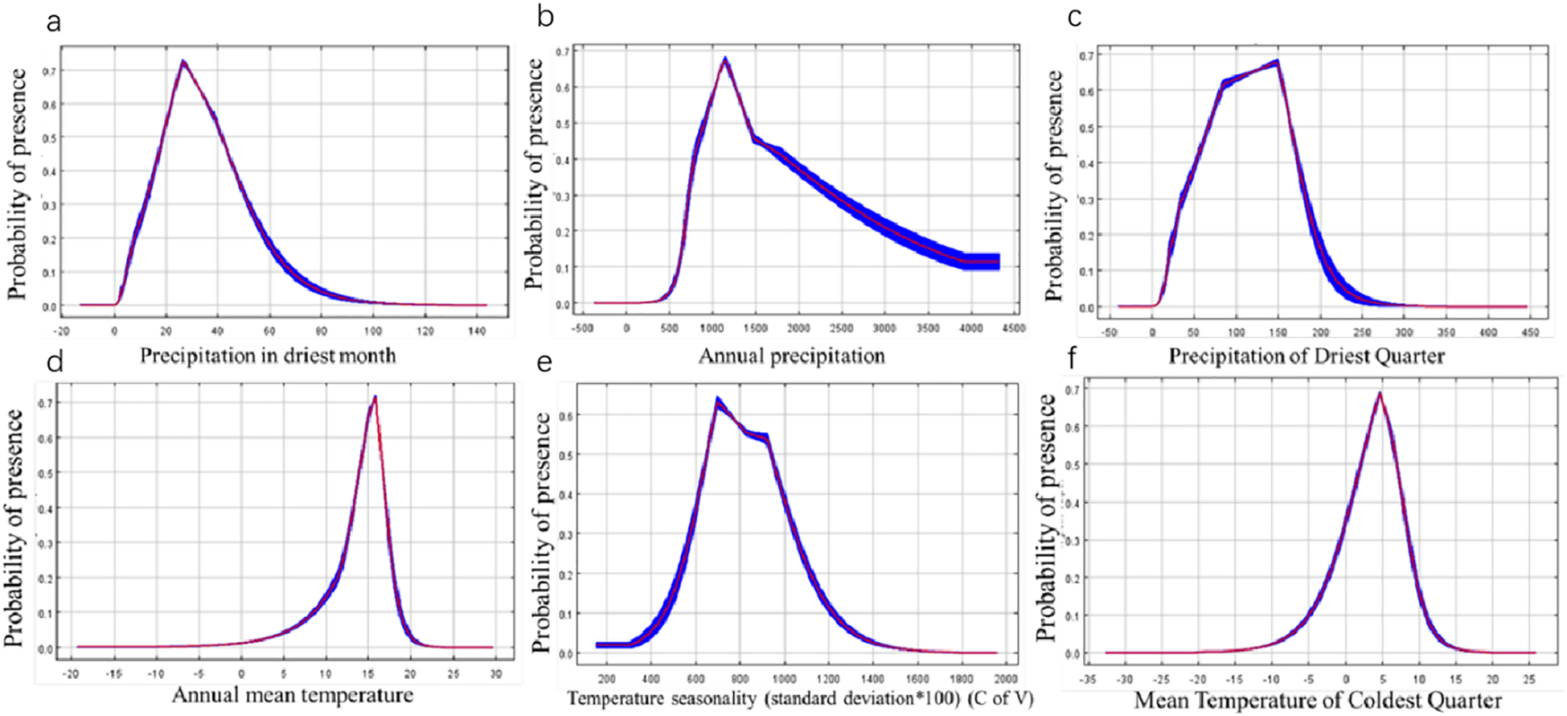
Response curves for dominant environmental factors. **(A)** Precipitation of Direst Month. **(B)** Annual Precipitation. **(C)** Precipitation of Direst Quarter. **(D)** Annual Mean temperature. **(E)** Temperature Seasonality (standard deviation* 100). **(F)** Mean temperature of Coldest Quarter.

In order to better grow *P. heterophylla*, further investigations were carried out to obtain the optimum habitat values of the above six key environmental variables (Bio14, Bio12, Bio17, Bio1, Bio4, Bio11). A presence probability greater than 0.5, the same range with optimum habitat, was used to estimate the range of the optimum habitat value for each variable. Maximum entropy modeling for the current period showed increasing *P. heterophylla* occurrence with higher precipitation of driest month (variable Bio14), with the optimal precipitation ranging from 18.8116 to 41.2911 mm (**Figure 4A**). Probability of the plant’s occurrence exceeded 60% when annual precipitation (Bio12) was between 906 and 1394 mm (**Figure 4B**); precipitation of driest quarter (Bio17), between 64 and 164 mm (**Figure 4C**); and annual mean temperature (Bio1), between 13 and 16°C (**Figure 4D**). The optimal range of temperature seasonality (Bio4) was 651-935 (**Figure 4E**), while that of mean temperature of coldest quarter (Bio11) was 2.04-6.84°C (**Figure 4F**).

### 3.3 Potentially suitable habitats of *P. heterophylla* under current climate scenarios

**Figure 5:**
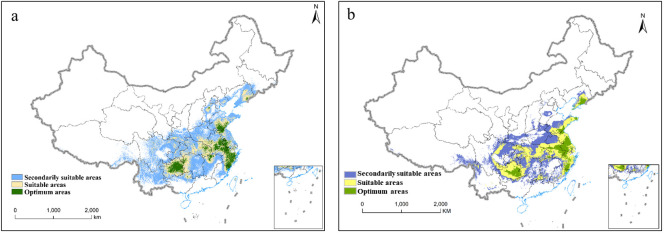
The potential suitable habitats of *P. heterophylla* in China under current scenarios. **(A)** The potential suitable habitats of *P. heterophylla* in China(55 environmental variables). **(B)** The potential suitable habitats of *P. heterophylla* in China(19 environmental variables).

The MaxEnt was used to simulate the distribution map of suitable areas for *P. heterophylla* in the current period (**Figure 5**). Using 55 environmental variables, our established model accurately predicted the predominant distribution of *P. heterophylla* optimum habitats in central Guizhou, northeastern Fujian, eastern Anhui, western Jiangsu, and southern Shandong (**Figure 5A**). Additionally, Using 19 climatic factor, we model and predict the optimum habitats of *P. heterophylla* were mainly distributed in the southwestern region (Guizhou) and eastern regions (Anhui, Zhejiang, Fujian, Jiangsu) of China (**Figure 5B**). The similarity in the prediction of suitable habitat based on 55 ecological factors and 19 climatic factors further supports the notion that climate factors predominantly influence the optimal growth conditions for *P. heterophylla*.

### 3.4 Model prediction of *P. heterophylla* distribution under future climate scenarios

**Figure 6:**
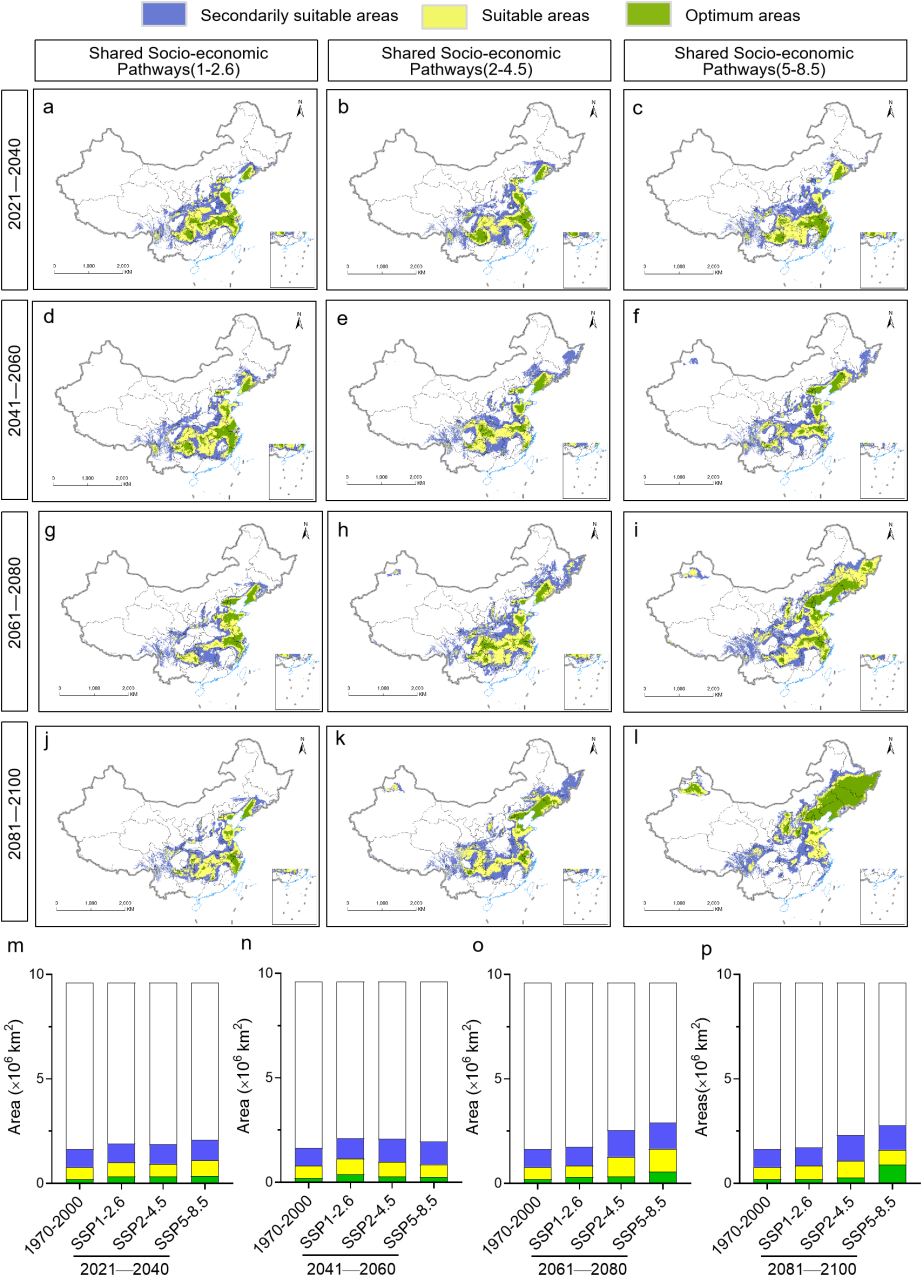
Evolution of the location and area of habitat suitable for *P. heterophylla* in China from 2021 until 2100. **(A-L)** Distribution of suitable areas. **(M-P)** Comparison of surface areas of suitable habitats. The current distribution (1970-2000) is shown at the far left as a reference.

Maximum entropy modeling predicted that the area of habitat suitable for *P. heterophylla* would increase in the future, but this increase would be primarily in suitable and secondarily suitable habitats (**Figure 6**). Under the SSP1-2.6 scenario, the area of habitat showing low suitability for the plant is predicted to increase by 0.033×10^6^-0.103×10^6^ km^2^ during the four periods that we analyzed, while the area of optimal habitat is predicted to decrease by 0.004×10^6^ km^2^ from 2081 to 2100. Under the SSP2-4.5 scenario, in contrast, the area of habitat showing low suitability is predicted to increase by 0.069×10^6^-0.403×10^6^ km^2^ and the area of optimal habitat by 0.078×10^6^-0.135×10^6^ km^2^ during the four periods. Similarly, under the SSP5-8.5 scenario, the area of habitat showing low suitability is predicted to increase by 0.117×10^6^-0.379×10^6^ km^2^ and the area of optimal habitat by 0.059×10^6^-0.691×10^6^ km^2^. In both of these scenarios, the largest increase in habitat of low suitability is predicted to occur in 2061-2080, and the largest increase in optimal habitat is predicted to occur in 2021-2040.

### 3.5 Multivariate environment similarity surface and most dissimilar variable analysis

**Figure 7:**
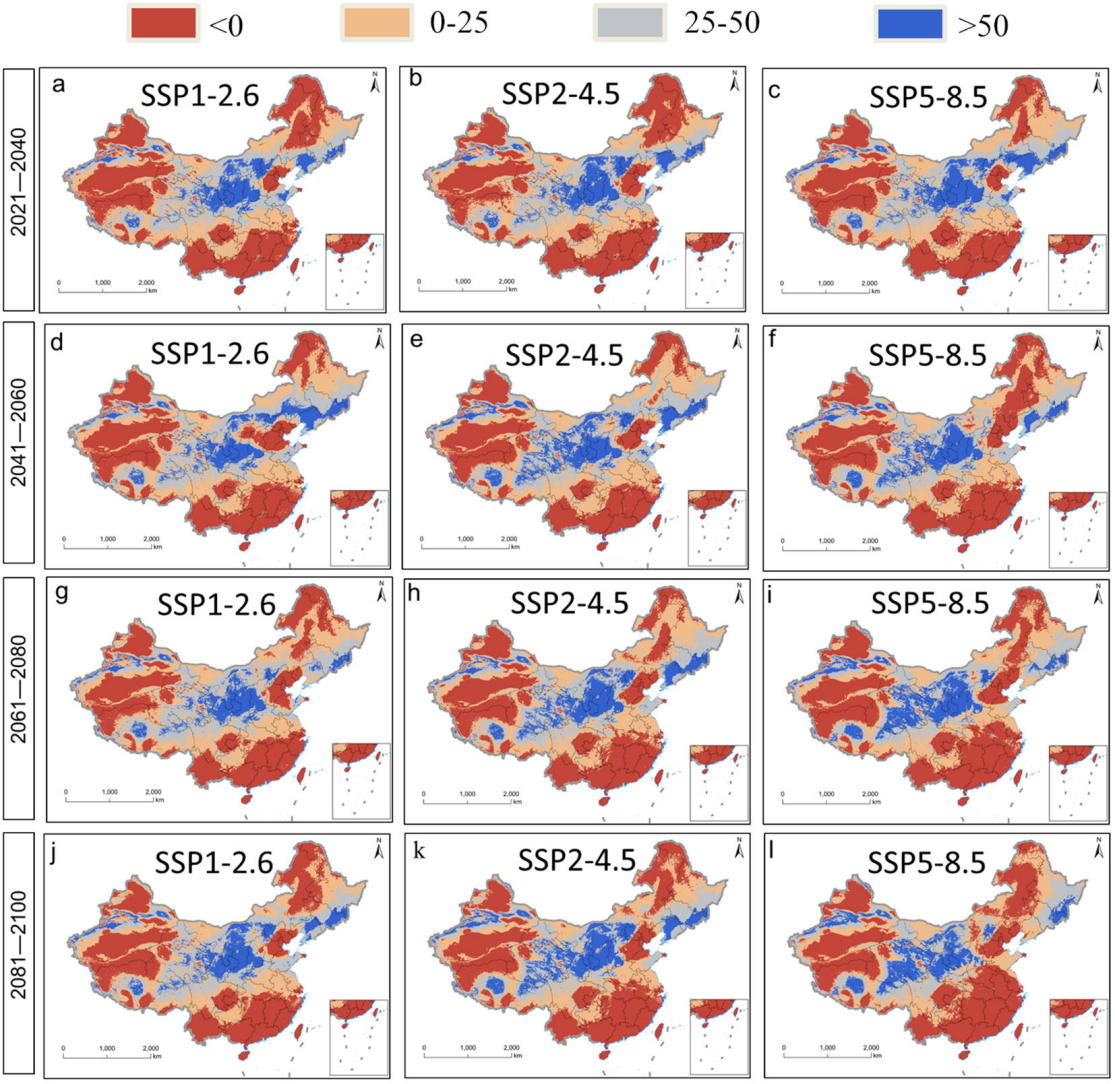
Multivariate environmental similarity surface [MESS, **(A–L)**] analysis for *P. heterophylla.*.

**Figure 8:**
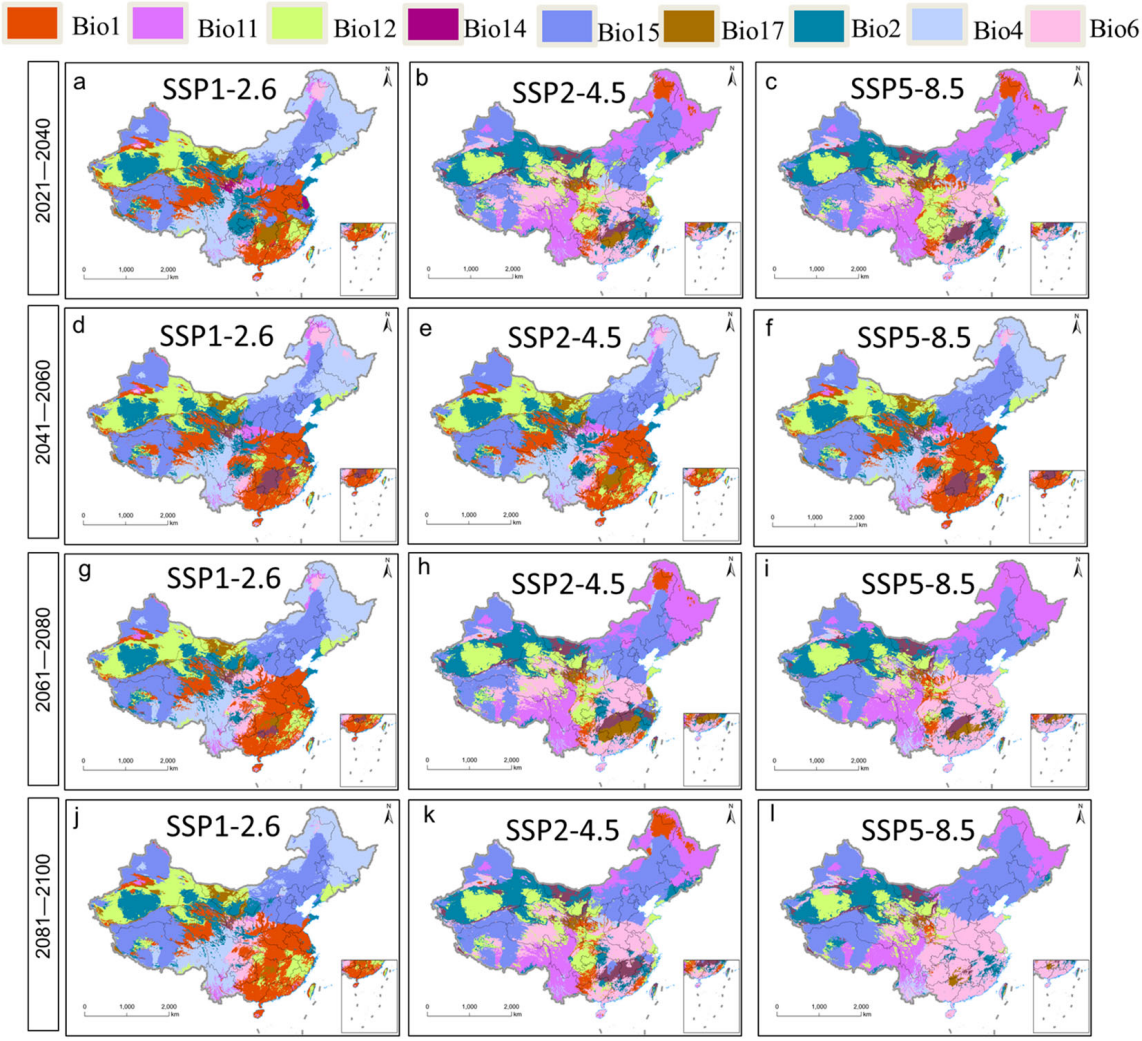
Most dissimilar variable [MoD, **(A–L)**] analysis for *P. heterophylla*.

Future average multivariate similarities under different climate change scenarios ranged from 8.40 to 24.98 (**Figures 7A–L**). Scenario SSP1-2.6 showed the highest similarity and weakest climate anomaly, while scenario SSP5-8.5 showed the lowest similarity and strongest anomaly. The variables predicted to be most influential on future plant distribution, regardless of scenario, were annual mean temperature, minimal temperature in the coldest month, annual precipitation, precipitation in the driest quarter, and precipitation in the driest month (**Figures 8A–L**). The temperature-related variables were most influential over a much wider area than precipitation-related ones.

### 3.6 Relationships between main environmental variables and content of bioactive compounds in Pseudostellariae Radix

Relationships of the most influential ecological factors with levels of polysaccharides and heterophyllin B in Pseudostellariae Radix from 44 sites across China (**Table 3**) were explored using step regression. Two regression equations emerged, indicating significant correlations between content of polysaccharides or heterophyllin B with several ecological factor.

1. Y_1_=-79.109 + 2.461X_1_-3.435X_2_-8.893X_3_+4.307X_4_+0.126X_5_-0.016X_6_+0.422X_7_+0.04X_8_ (where Y_1_ is polysaccharide, X_1_ is Bio 6, X_2_ is Bio1, X_3_ is Bio2, X_4_ is Bio3, X_5_ is Bio 4, X_6_ is Bio 12, X_7_ is Bio 14, X_8_ is Bio 16. *R^2^* = 0.466, *P* = 0.039< 0.05)
2. Y_2_ = 0.013 + 0.001X_1_-0.009X_2+_0.002X_3_+0.0007578X_4_-0.0003139X_5+_0.0001476X_6_

**Table 3:** The contents of polysaccharide and Heterophyllin B in Pseudostellariae Radix from 44 sample plots.

NO.	Sampling place	polysaccharide content	Heterophyllin B content
1	Shouning County, Fujian 1	31.06 ± 2.62	ND
2	Shouning County, Fujian 2	32.43 ± 4.06	ND
3	Fuan City, Fujian 1	33.12 ± 3.06	ND
4	Fuan City, Fujian 2	27.24 ± 1.59	ND
5	Zherong County, Fujian 1	27.49 ± 2.56	ND
6	Zherong County, Fujian 2	31.31 ± 5.31	ND
7	Zherong County, Fujian 3	30.13 ± 1.16	ND
8	Zherong County, Fujian 4	28.95 ± 3.74	ND
9	Zherong County, Fujian 5	33.43 ± 4.50	ND
10	Zherong County, Fujian 6	33.45 ± 3.56	ND
11	Zherong County, Fujian 7	28.29 ± 1.97	ND
12	Xiapu County, Fujian	33.48 ± 0.47	ND
13	fuding city,Fujian	32.21 ± 2.03	ND
14	Dantu District, Jiangsu	33.42 ± 6.45	0.0242 ± 0.0019
15	Jurong city,Jiangsu 1	30.17 ± 2.22	0.0247 ± 0.0026
16	Jurong city,Jiangsu 2	32.44 ± 2.23	0.0298 ± 0.0036
17	Huoshan County, Anhui	27.42 ± 1.95	0.0219 ± 0.0016
18	Shucheng County, Anhui 1	31.14 ± 3.80	0.0261 ± 0.0019
19	Shucheng County, Anhui 2	31.14 ± 0.96	0.0343 ± 0.0024
20	Yu ‘an District, Anhui	37.04 ± 1.69	0.0222 ± 0.0027
21	Xuanzhou District, Anhui 1	22.61 ± 3.92	0.0204 ± 0.0007
22	Xuanzhou District, Anhui 2	29.63 ± 2.35	0.0188 ± 0.0015
23	Guangde County, Anhui 1	32.63 ± 1.22	0.0124 ± 0.0010
24	Guangde County, Anhui 2	27.65 ± 1.67	0.0065 ± 0.0008
25	Shibin County,Guizhou 1	38.07 ± 1.90	0.0118 ± 0.0017
26	Shibin County,Guizhou 2	35.22 ± 3.06	0.0161 ± 0.0007
27	Shibin County,Guizhou 3	39.42 ± 1.27	0.0231 ± 0.0006
28	Shibin County, Guizhou 4	32.74 ± 1.48	0.0248 ± 0.0026
29	Shibin County, Guizhou 5	25.87 ± 1.74	0.0122 ± 0.0016
30	Shibin County, Guizhou 6	27.99 ± 2.18	0.014 ± 0.0013
31	Qianxi County, Guizhou	29.75 ± 3.21	0.0258 ± 0.0011
32	Yuping County, Guizhou	30.98 ± 3.51	0.0238 ± 0.0019
33	Qingzhen City, Guizhou	33.21 ± 2.13	0.0111 ± 0.0011
34	Danzhai County, Guizhou	30.64 ± 2.92	0.0267 ± 0.0013
35	Pingtang County, Guizhou	35.28 ± 2.82	0.0171 ± 0.0011
36	Huaxi District, Guizhou	29.09 ± 1.74	0.0188 ± 0.0027
37	Zhenyuan County, Guizhou	28.03 ± 2.10	0.016 ± 0.0026
38	Fuquan City, Guizhou	29.93 ± 4.01	0.0208 ± 0.0007
39	Yuqing County, Guizhou	21.55 ± 2.35	0.0188 ± 0.0015
40	Huangping County, Guizhou	30.93 ± 4.50	0.0143 ± 0.0017
41	Linshu County, Shandong	32.2 ± 4.34	0.0123 ± 0.0007
42	Luozhuang Distric,Shandong	32.14 ± 1.53	0.0124 ± 0.0012
43	Yinan County,Shandong	28.68 ± 1.15	0.0147 ± 0.0022
44	Hedong District, Shandong	32.73 ± 2.06	0.0127 ± 0.0016

Values are mean ± SD of three replicates.”ND” means not detected or below the quantitative limit.

(where Y_2_ is Heterophyllin B content, X_1_ is Bio6, X_2_ is Bio2, X_3_ is Bio3, X_4_ is Bio4, X_5_ is Bio12, X_6_ is Bio 16. *R^2^* = 0.467, *P* = 0.000< 0.05.)

These equations show that the contents of two active compounds are mainly related to temperature. The ecological factors that impact polysaccharides include Bio 6, Bio1, Bio2, Bio3, Bio4, Bio12, Bio14 and Bio16. Among these factors, the primary influencers of polysaccharides are Min Temperature of Coldest Month (bio6), Annual Mean Temperature (bio1), Mean Diurnal Range (bio2), and Isothermality (Bio2/Bio7) (*100) (bio3). The ecological factors that influence Heterophyllin B also encompass Bio 6、Bio2、Bio3、Bio4、Bio12 and Bio16. Among these factors, the key determinants affecting Heterophyllin B are Min Temperature of Coldest Month (Bio6), Mean Diurnal Range (Bio2), and Isothermality (Bio2/Bio7) (*100) (bio3).

### 3.7 Predicted distribution of polysaccharide content in the future

**Figure 9:**
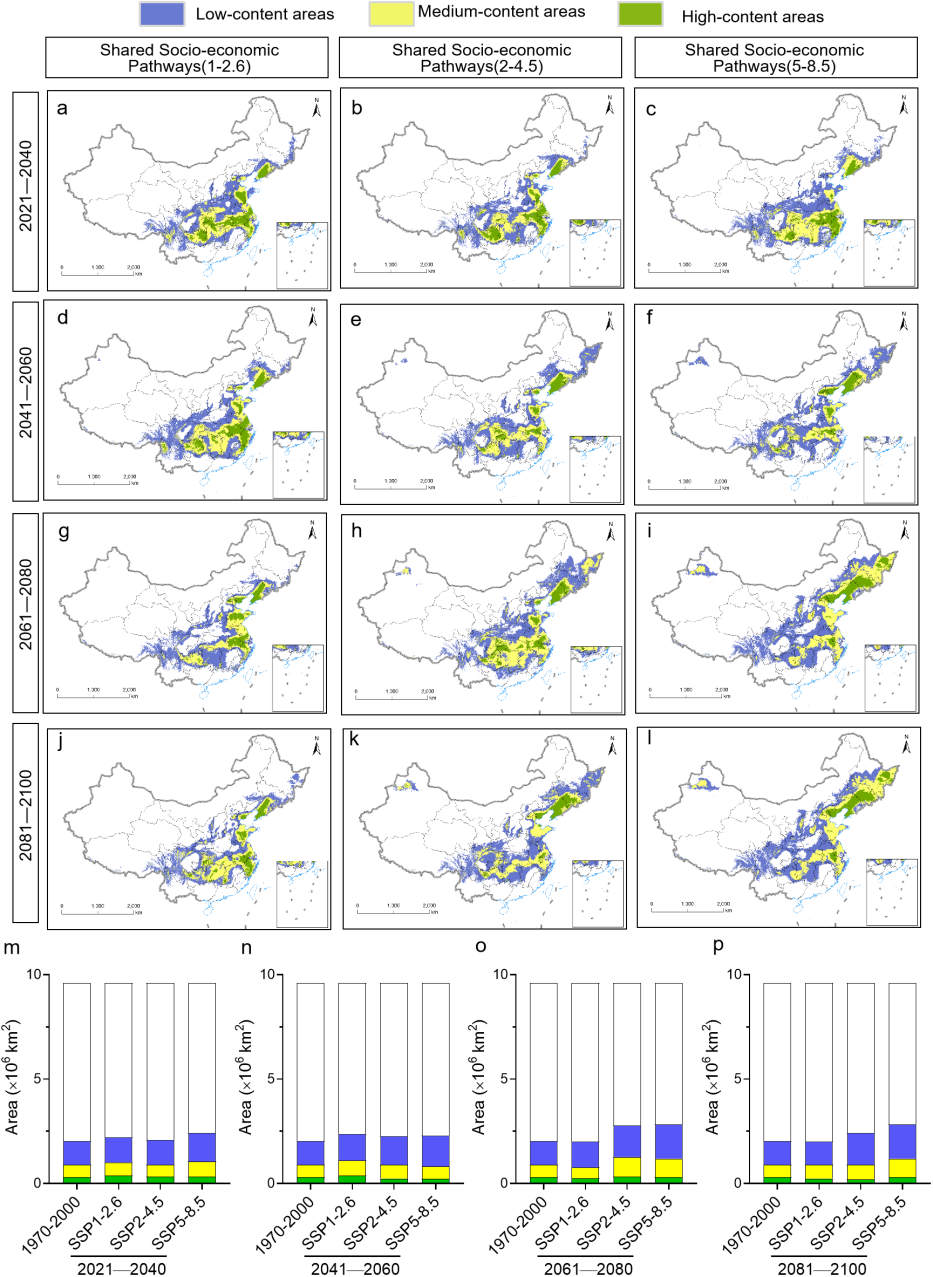
Predicted distribution of polysaccharide content in *P. heterophylla* in China under different climate scenarios during different time periods from 2021 to 2100. Sites were colored according to whether the plants at those sites were predicted to contain low, intermediate, or high content of polysaccharides. **(A-L)** Geographic distribution. **(M-P)** Comparison of surface areas. The current distribution (1970-2000) is shown at the far left as a reference.

The spatial analysis function of ArcGIS was used to explore the future distribution of polysaccharide content of Pseudostellariae Radix under different climate change scenarios (**Figures 9A–L**). Regardless of the scenario, high polysaccharide content was predicted for *P. heterophylla* in Hebei, Shaanxi, Shandong, Jiangsu, Anhui, Zhejiang, Jiangxi, Henan, northeast Hubei, northwest Hunan, and Sichuan (**Figures 9A–L**). These areas are predicted to be larger than the current distribution (**Figures 9M–P**). Although there were variations in the areas with high polysaccharide content across different time periods under future climate scenarios, the total area of these regions showed minimal disparity.

### 3.8 Predicted distribution of heterophyllin B content in the future

**Figure 10:**
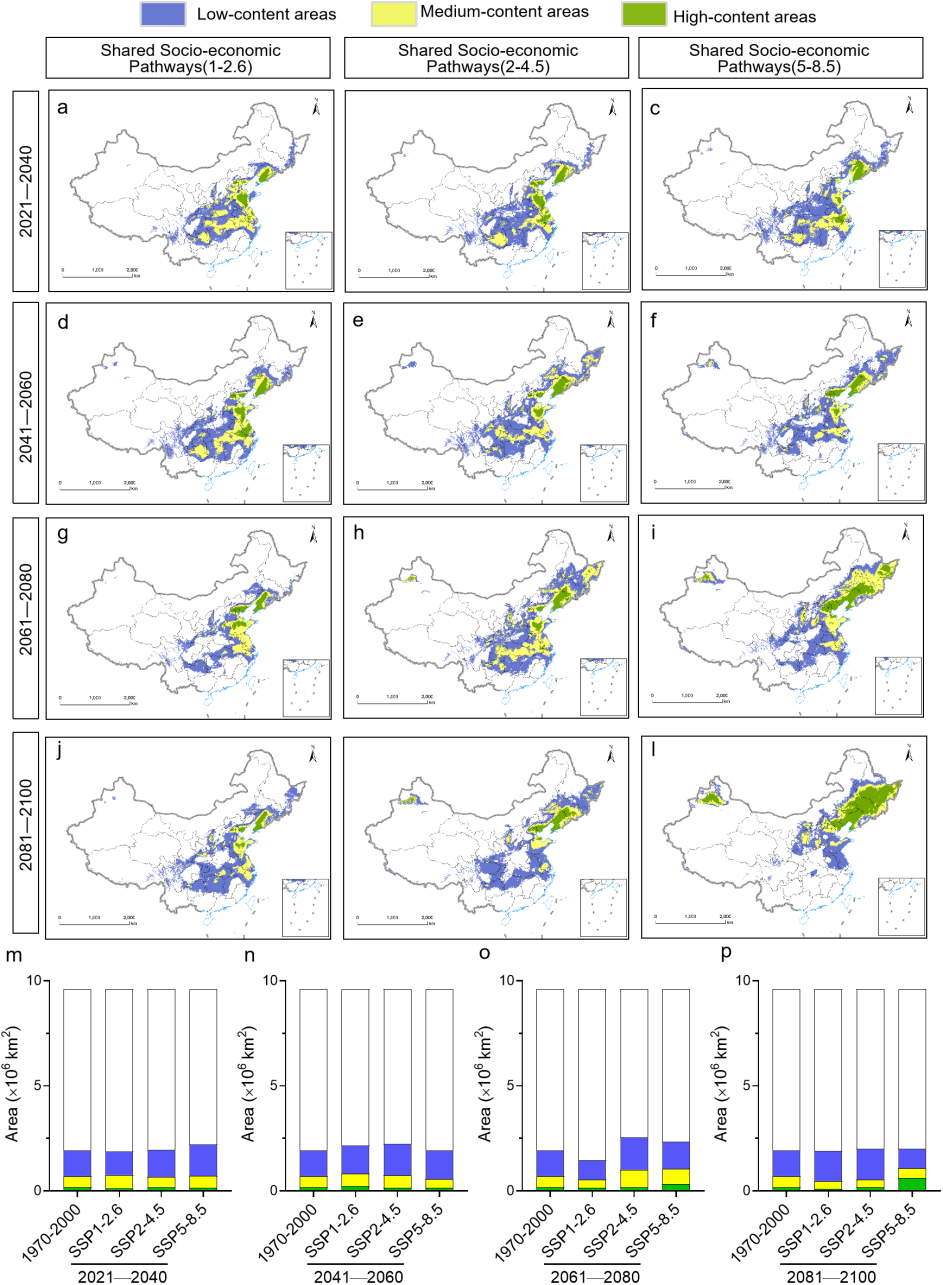
Distribution of Heterophyllin B content in *P. heterophylla* in China under different future climate scenarios during different time periods from 2021 to 2100. **(A-L)** Geographic distribution. **(M-P)** Surface areas during each time period under each climate scenario. For comparison, the surface area is shown for the current *P. heterophylla* distribution (1970-2000).

In contrast, high heterophyllin B content was predicted for different regions depending on the climate change scenario and time period. High content was located mainly in Shandong, Guizhou, western Hunan and Hubei under the SSP1-2.6 scenario, while it was located mainly in Hubei and Shaanxi under the SSP2-4.5 scenario for the period from 2041 to 2060. In contrast, high content was located mainly in Henan and northern Hubei under the SSP5-8.5 scenario for the period from 2081 to 2100. This modeling suggests that the area covered by high heterophyllin B content will increase in the future, especially under the SSP5-8.5 scenario (**Figure 10**).

### 3.9 Trends in migration of the centroid of suitable *P. heterophylla* habitat and the centroid of levels of bioactive components

**Figure 11:**
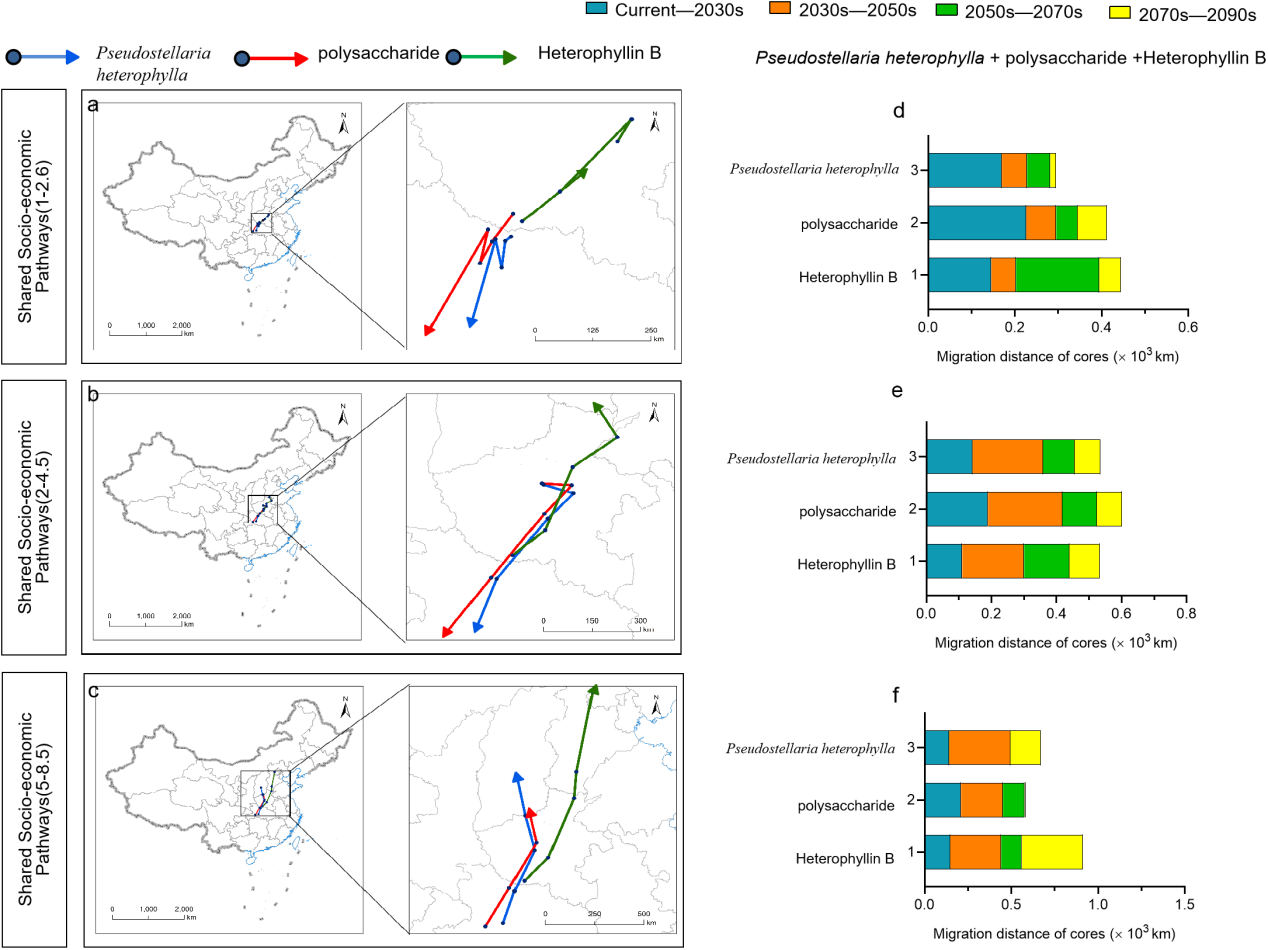
Geographical directions **(A-C)** and distances **(D-F)** of predicted migration of the centroids of habitat suitable for P. heterophylla, for P. heterophylla high in polysaccharides and for P. heterophylla high in heterophyllin B within China, under three climate change scenarios.

In this study, There were large differences in suitable range shift locations of *P. heterophylla* under different climate scenarios. We found that the center of mass suitable *P. heterophylla* habitat and the center of mass levels of bioactive components are not in the same area. Under the future climate scenarios SSP1-2.6, SSP2-4.5, the migration direction of the center of mass in the suitable area is southwest, which is located in the north of Hubei Province. Under the future climate scenario SSP5-8.5, the direction of the center of mass migration in the suitable area is the north. Under the three Climate Scenarios, Migration with a migration distance of 295 ~ 669 km. The migration trend of polysaccharide centroid is consistent with that of suitable area, and the migration distances are 411 km, 601 km and 585 km. The center of mass of Heterophyllin B content was predicted to shift to the north from the current area in the future by 445 km under SSP1-2.6, 534 km under SSP2-4.5, and 912 km under SSP5-8.5. Under the future climate scenarios SSP1-2.6 and SSP2-4.5, the center of mass Heterophyllin B and the center of mass of the suitable area move in different directions. Under the future climate scenario SSP5-8.5, the migration direction of the center of mass of the suitable area aligns with that of polysaccharide centroid and Heterophyllin B centroid (**Figure 11**).

## 4 Discussion

Due to variations in research fields and objects, the utilization of diverse models can potentially influence the predictive efficacy of the model. Therefore, it is crucial to choose an appropriate model and optimize the model before making predictions on the ecological suitability of *P. heterophylla* and changes in areas with a high bioactive component content. With the progress of scientific research, a variety of technical models have been employed for species distribution prediction, including GAM (Gaira et al., 2011), GLM (Zilko et al., 2020), MaxEnt (Tang et al., 2021), MARS (Xia et al., 2019), RF (Asamoah et al., 2024), Genetic Algorithm for Rule-Set Production model (GARP) (Yang A. et al., 2020) and Climate Change Experiment model (Climex) (Souza et al., 2023) among others. The 9 models employed for screening in this study possess distinct advantages. ANN and RF offer comprehensive insights into habitat distribution, demonstrating robust spatial performance. FDA and CTA are advantageous for statistical analysis as they do not rely on assumptions regarding the relationship between response variables and prediction variables, effectively handle nonlinear relationships, and provide powerful analytical capabilities. GAM, GLM, and MARS are regression algorithms that exhibit unique strengths in handling different distribution forms based on various dependent variables. MaxEnt demonstrates remarkable predictive accuracy even in scenarios with limited sample sizes. SRE is distinguished by its simplicity and user-friendly nature. AUC and TSS are widely employed metrics for assessing niche model performance, with a higher value indicating greater accuracy of the model (Wang et al., 2019b). It was generally believed that the model performance was poor when AUC value was 0.5-0.7, moderate when AUC value was 0.7-0.9, and good when AUC value was greater than 0.9 (Liu et al., 2011). The results demonstrated that the MaxEnt exhibited a superior performance with an AUC value of 0.969 and a TSS value of 0.845, surpassing all other models tested in this study. These findings unequivocally establish the MaxEnt as the optimal choice for *P. heterophylla* modeling.

The MaxEnt has been widely used in ecology, conservation biology, evolutionary biology, and invasive species management (Phillips et al., 2006). The accurate prediction of the potential niche with distribution of species requires rational data collection and precise parameter optimization. This study established 8 numerical regularization multipliers ranging from 0.5 to 4 and adopted 9 characteristic combinations, namely “L”, “LQ”, “H”, “LQH”, “LQHP”, “LQHPT”, “QHP”, “QHPT,” and “HPT”, These combinations were used to evaluate a total of 72 parameter combinations in the ENMeval package in R software. The results of this study indicated that the MaxEnt optimized with the parameters FC = LQH and RM =1 was found to be optimal (**Figure 3**). In general, with a large sample capacity, the model’s prediction accuracy is high. The MaxEnt can be successfully predicted with a sample size of ≥5, resulting in more accurate predictions (Pearson et al., 2007). The R language was used to identify and select 330 geographical distribution points, and relevant factors for modeling were determined through Pearson correlation analysis. Through meticulous data sorting, rigorous model selection, and comprehensive parameter optimization, we ensure the predictive accuracy of the established Maxent for *P. heterophylla* distribution.

The distribution of most plants is mainly affected by temperature and precipitation, among all environmental factors, according to reports (Liu et al., 2023; Shi et al., 2023; Liao et al., 2024). Temperature and precipitation variations at different latitudes and longitudes impact the quantity and distribution of plant populations. Consistently, in our results, Climatic factors were generally much more important than soil, topographic and solar radiation variables for the distribution of *P. heterophylla*. All of the top 8 ecological factors that exert the most influence are connected to variations in temperature and precipitation (**Supplementary Table S5**). Our analysis identified temperature and precipitation as perhaps the most important environmental determinants of *P. heterophylla* distribution. The result of this study also agrees with the growing habits of *P. heterophylla*, that it thrives in warm and humid environments, exhibits cold tolerance, shows sensitivity to high temperatures, and undergoes dormancy at 30 degrees Celsiu (Xiao et al., 2013). The model indicated that Guizhou in southwest China, as well as Fujian and Anhui in east China, were identified as suitable distribution regions for *P. heterophylla*, which are indeed among the centers of production of Pseudostellariae Radix in the country. The coincidence of temperature and rainfall in certain regions of these provinces may account for the favorable growth conditions for *P. heterophylla*. In Guizhou, the annual average temperature is 14.8 °C and the annual precipitation is 987 mm, compared to 17 °C and 1373 mm in Fujian.

The distribution pattern of plants is expected to undergo significant changes in response to future climate change. Mounting evidence suggests that the global mean temperature is on the rise and precipitation also changes (Ma et al., 2024). In response to climate change scenarios, a logical consequence is the spatial redistribution of species towards higher elevations, taking advantage of increased precipitation and cooler temperature (Anderson and Wadgymar, 2019; Hamid et al., 2019; Zu et al., 2021). This process facilitates the development of plants that are better adapted to the prevailing conditions. Here, we present compelling evidence indicating that ongoing climate change will lead to the migration of suitable habitats for *P. heterophylla* towards the southwest and north, resulting in an expanded range of favorable areas. In other words, habitats suitable for *P. heterophylla* will shift toward higher latitudes and high altitude, as predicted for numerous plant species as temperatures concentrations rise. According to reports, there will be a shift in the suitable habitat areas of medicinal plants from southern to northern China, resulting in a significant loss of suitable habitat areas for southern China, it is projected that the western regions of China will encompass significantly larger suitable habitat areas in the future (Xia et al., 2022).

Climate change may lead to the extinction of some regions or local species. At the same time, some ecosystems can be replaced by other ecosystems as suitable habitats (Pereira et al., 2016) and the distribution areas with high content of chemical composition are constantly replaced. Our findings suggest that the future migration trends of *P. heterophylla* suitable habitats differ from those with high heterophyllin B content, but they resemble those associated with high polysaccharide content. Heterophyllin B is a secondary metabolite. The presence of ecological stressors that are unfavorable for plant growth can induce the biosynthesis of secondary metabolites (Sun et al., 2023; El-Mahdy et al., 2024). This also indicates that the content of secondary metabolites is low in the suitable growth areas of plants. For example, the content of secondary metabolites in highly suitable habitats of *Panax notoginseng* is low. Polysaccharide, being a primary metabolite (Honório et al., 2024) exhibits a proportional increase in content with the size of *P. heterophylla* tubers (Kang et al., 2014) thereby demonstrating its consistent response to environmental suitability.

The distribution of plants is shaped not only by climatic factors but also by non-biological elements such as topography, soil, and human activities, including socioeconomic development, human intervention, and policie (Courchesne et al., 2020; Miron et al., 2021; Zou et al., 2023; Pironon et al., 2024). The MaxEnt accurately simulates the suitable range of *P. heterophylla*. The results can provide a scientific basis for *P. heterophylla* to formulate conservation strategies and adapt to future climate change. It is crucial to recognize that the distribution region in this study solely represents the appropriate range of *P. heterophylla* based on climate factors, and it does not necessarily imply complete congruence with the actual distribution area in future scenarios, its distribution and migration are influenced by a combination of numerous factors. The present study still possesses several limitations that necessitate attention. For instance, it did not account for biotic factors such as species interactions and human activities. Future studies could improve the accuracy of these predictions by incorporating additional factors, such as human activities, soil conditions, land use dynamics, and species interactions. This will facilitate a more comprehensive and precise assessment of the distribution patterns and changes in *P. heterophylla*.

## 5 Conclusions

In this study, the model and default parameters were screened and optimized, resulting in the identification of FC=LQH and RM=1 as the optimal MaxEnt after parameter optimization. The average value of the area under the ROC curve (AUC) was 0.969, indicating that the prediction accuracy of the MaxEnt for the potential habitable zone of *P. heterophylla* was high. We developed a methodology to assess the impact of climate factors on *P. heterophylla* quality, utilizing bioactive components and MaxEnt in conjunction with database records, sampling techniques, and component analysis. Using the MaxEnt, the climate factors affecting the suitable distribution area of *P. heterophylla* were screened as precipitation of driest month and driest quarter, annual precipitation, annual mean temperature, temperature seasonality, and mean temperature of coldest quarter. Under current climate conditions, suitable habitats of *P. heterophylla* were mainly distributed in the southwestern region (Guizhou) and eastern regions (Anhui, Zhejiang, Fujian, Jiangsu) of China. As the climate continues to change, by the 2090s, suitable habitats and distribution area of high heterophyllin B content under SSP5-8.5 climate scenarios will increase significantly, other periods and climate scenarios have little difference in area. The future migration trends of suitable habitats differ from those with high heterophyllin B content, but they resemble those associated with high polysaccharide content. Our results imply that artificial migration may be important for ensuring sustainable cultivation of *P. heterophylla*. The analysis method proposed by us offers a valuable reference for the conservation of Chinese herbal medicine resources and the selection of cultivation areas, while also providing a novel perspective for assessing the impact of ecological factors on the quality of Chinese herbal medicine.

## Data Availability

The original contributions presented in the study are included in the article/**Supplementary Material**. Further inquiries can be directed to the corresponding authors.
