# Supplementary material for: Simulation of Pseudostellaria heterophylla distribution in China: assessing habitat suitability and bioactive component abundance under future climate change scenariosplant components
Source: Front Plant Sci. 2024 Dec 4;15:1498229. doi: 10.3389/fpls.2024.1498229 (PMC11653070; doi:10.3389/fpls.2024.1498229)
Supplement: Supplementary file 1 [file DataSheet1.docx]

Table.S1.Detailed description of data analysis operation method

| Data preparation | Data source | Data analysis |
| --- | --- | --- |
| (A) Obtaining and processing occurrence data | we collected data on 1521(The deadline is 2023.) occurrence records of *P. heterophylla* distribution from the National Specimen Information Infrastructure (http://www.nsii.org.cn/2017/home.phpNSII), Chinese Virtual Herbarium (https://www.cvh.ac.cn/), Plant Photo Bank of China (http://ppbc.iplant.cn/), and the Chinese National Knowledge Infrastructure database. | The R software package “spThin” was used to remove the samples that clustered within 10 km to reduce the sampling deviation impact,remove the natural records, and delete redundant data. |
| 1. Obtainingand processing environmental data | downloaded from the Worldclim 2.1 database (http://www.worldclim.org/) for the following periods: last interglacial period, last glacial maximum, mid-Holocene, the current period and the future. Data had a spatial resolution of 30′′ (approximately 1 km^2^). | Using the conversion tool in ArcMap software of ArcGis 10.4, the downloaded layers are converted into asc format files, and all the layers are projected into WGS84 geographic coordinate system, and the cutting tool is used to cut them to include only China area to reduce the running time of the model. |
| 1. Model   screening |  | The Biomod2 package in R language is used to model and compare the niche models of ninespecies distribution, including surface range envelope) model (SRE), Random forest algorithm (RF), Flexible discriminant analys, FDA), Classification tree analysis (CTA), generalized boosted regression models (GAM), Artificial neural network, ANN), General liner model (GLM), multivariate adaptive regression splines (MARS),and Maximum entropy model (MaxEnt). ENMeval optimized feature class setting and β regularization multiplier are used, and Biomod2 default model setting is used for other algorithms. |
| (D)Model parameter optimization |  | ENMeval and biomod2 data packets in R language are used to optimize two parameters, namely Regularization multiplier (RM) and Feature combination (FC), and the "regularization multiplier" and "feature combination" modules in ENMeval package in R language are used to reduce the model complexity and improve the model accuracy. The value of the regularized multiplier is between 0.5 and 4, and each time it is increased by 0.5, a total of 7 times of frequency multiplication is increased. Where l is Linear, q is Quadratic, h is fragmented, p is Product and t is Threshold. Nine feature combinations are used: L, H, LQ, HPT, QHP, LQH, LQHP, QHPT, LQHPT. ENMeval data package tests the above 72 parameter combinations. Finally, the difference between training and testing AUC (AUCdiff) and 10% test omission rate (or 10pct) are used to test the fitting degree of the model to species distribution points. Delta. AICc in Akaike Information Criterion Correction (AICC) model evaluates the complexity and fitness of different parameter combinations, and finally selects the best model parameter combination with the lowest AICC (delta.AICc = 0) for modeling. |
| (e)Current and future potential habitat evaluations |  | The regionalization model demonstrated the distribution of suitable habitats for *P. heterophylla* ArcGIS 10.4.1was used to transform the results, and raster data were output. The attributes of the setting layer were defined using the “reclassify” tool in ArcGIS based on the natural breakpoint method was used to classify the results and obtain a gradient classification for the suitable habitats. suitability as unsuitable (fitness value, 0-0.1), secondarily suitable (0.1-0.25), suitable (0.25-0.5), or optimal (0.5-1). The change in suitable habitat area was calculated by counting the number of grids at all suitability levels. Spatial units with a species existence probability ≥ 0.1 were regarded as suitable areas, and spatial units with a species existence probability ˂ 0.1 were regarded as unsuitable areas . |
| (f)Model construction of index components and ecological factors |  | Based on ArcGIS, the ecological factor values of 44 Radix Pseudostellariae producing areas were extracted. Using SPSS, the content of polysaccharide and cyclophosphamide B and the data of ecological factors were gradually regressed, and the relationship model between polysaccharide and Heterophyllin B and the main ecological factors was constructed. Through the relationship model, the relationship between the content of index components of Pseudostellaria heterophylla and ecological factors was discussed. |
| (G)Spatial distribution analysis of index component content |  | Using the grid calculation function of ArcGIS software, the ecological factors are taken as independent variables to the spatial distribution map of the component content constructed by the relationship model between the index component content and ecological factors. Based on the relationship model between the index component of Pseudostellaria heterophylla and climate factors, the spatial distribution of the index component content of *Pseudostellaria heterophylla* in China is estimated by using the grid calculation function of ArcGIS software. |

Table.S2. Speciation records of *Pseudostellaria heterophylla*

| NO. | longitude | latitude | NO. | longitude | latitude | NO. | longitude | latitude |
| --- | --- | --- | --- | --- | --- | --- | --- | --- |
| 1 | 119.5521 | 27.2567 | 111 | 108.3428 | 26.8811 | 221 | 119.2178 | 30.9645 |
| 2 | 119.5730 | 27.3816 | 112 | 108.3428 | 27.0061 | 222 | 119.2178 | 31.6311 |
| 3 | 119.6698 | 27.1846 | 113 | 108.3428 | 28.8811 | 223 | 119.2178 | 31.6728 |
| 4 | 119.7018 | 27.2248 | 114 | 108.3845 | 27.1311 | 224 | 119.2178 | 31.7561 |
| 5 | 119.7767 | 27.2468 | 115 | 108.4261 | 25.6728 | 225 | 119.2178 | 34.2978 |
| 6 | 119.8198 | 27.2754 | 116 | 108.4261 | 26.2145 | 226 | 119.2178 | 35.7561 |
| 7 | 119.9031 | 27.2032 | 117 | 108.4678 | 26.3811 | 227 | 119.2595 | 31.6311 |
| 8 | 119.9288 | 27.1418 | 118 | 108.8428 | 26.3811 | 228 | 119.2595 | 31.6728 |
| 9 | 119.8598 | 27.1223 | 119 | 108.9261 | 27.2561 | 229 | 119.2595 | 31.7145 |
| 10 | 119.8689 | 27.0502 | 120 | 109.0095 | 26.9645 | 230 | 119.2595 | 31.7978 |
| 11 | 119.9722 | 27.2421 | 121 | 109.0511 | 26.9645 | 231 | 119.2595 | 31.9228 |
| 12 | 120.0046 | 27.1874 | 122 | 109.1761 | 26.9645 | 232 | 119.2595 | 32.0478 |
| 13 | 120.0356 | 27.2565 | 123 | 109.2595 | 25.9645 | 233 | 119.3011 | 31.7145 |
| 14 | 119.3121 | 32.1681 | 124 | 109.3845 | 31.5478 | 234 | 119.3011 | 31.7978 |
| 15 | 119.2863 | 31.7178 | 125 | 109.5095 | 31.8811 | 235 | 119.3011 | 31.9228 |
| 16 | 119.2683 | 31.6809 | 126 | 110.3428 | 31.5478 | 236 | 119.3011 | 34.6728 |
| 17 | 119.1887 | 31.3618 | 127 | 110.5928 | 27.9228 | 237 | 119.3428 | 27.0061 |
| 18 | 116.9111 | 31.3784 | 128 | 111.0095 | 27.1728 | 238 | 119.3428 | 34.6728 |
| 19 | 117.0169 | 31.4515 | 129 | 111.9261 | 25.5895 | 239 | 119.3845 | 27.3811 |
| 20 | 116.3838 | 31.7395 | 130 | 112.3428 | 26.9645 | 240 | 119.3845 | 30.2145 |
| 21 | 119.5363 | 30.8157 | 131 | 112.8428 | 27.2561 | 241 | 119.3845 | 30.7978 |
| 22 | 118.8000 | 30.8052 | 132 | 113.3845 | 31.6728 | 242 | 119.3845 | 30.9228 |
| 23 | 118.7949 | 30.8637 | 133 | 113.6345 | 35.3395 | 243 | 119.3845 | 31.9228 |
| 24 | 119.2494 | 30.9200 | 134 | 113.8011 | 35.4645 | 244 | 119.4261 | 30.3395 |
| 25 | 118.0884 | 26.9878 | 135 | 113.8011 | 40.5895 | 245 | 119.4678 | 34.7145 |
| 26 | 105.9230 | 26.9885 | 136 | 113.8428 | 22.6728 | 246 | 119.5095 | 29.4645 |
| 27 | 109.1167 | 27.4090 | 137 | 113.8428 | 40.5895 | 247 | 119.5095 | 35.4228 |
| 28 | 108.0220 | 27.2158 | 138 | 113.9678 | 35.2978 | 248 | 119.5928 | 31.2145 |
| 29 | 108.1773 | 27.0689 | 139 | 114.1761 | 30.5478 | 249 | 119.5928 | 31.3395 |
| 30 | 106.2601 | 26.7667 | 140 | 114.9261 | 38.2145 | 250 | 119.5928 | 31.8811 |
| 31 | 107.8784 | 26.1817 | 141 | 114.9261 | 38.5061 | 251 | 119.6761 | 27.3395 |
| 32 | 107.2704 | 25.9278 | 142 | 115.3428 | 38.4228 | 252 | 119.6761 | 27.5895 |
| 33 | 107.9253 | 27.1402 | 143 | 115.7178 | 28.7145 | 253 | 119.7595 | 27.0478 |
| 34 | 106.5864 | 26.2742 | 144 | 115.7178 | 33.8811 | 254 | 119.8011 | 27.0061 |
| 35 | 108.3457 | 27.0058 | 145 | 115.7595 | 31.1728 | 255 | 119.8011 | 27.0895 |
| 36 | 107.4640 | 26.7735 | 146 | 115.8011 | 29.0478 | 256 | 119.8011 | 27.1311 |
| 37 | 108.2400 | 27.0647 | 147 | 115.8428 | 29.7145 | 257 | 119.8011 | 27.1728 |
| 38 | 107.9029 | 27.2128 | 148 | 115.8428 | 31.5895 | 258 | 119.8011 | 27.2145 |
| 39 | 108.2214 | 27.0433 | 149 | 116.0095 | 29.5478 | 259 | 119.8011 | 27.2561 |
| 40 | 107.8918 | 27.1316 | 150 | 116.1761 | 30.7145 | 260 | 119.8011 | 27.2978 |
| 41 | 118.5475 | 34.9143 | 151 | 116.1761 | 31.2145 | 261 | 119.8011 | 31.3395 |
| 42 | 118.3435 | 34.9210 | 152 | 116.2178 | 31.0895 | 262 | 119.8428 | 27.1311 |
| 43 | 118.5727 | 35.3567 | 153 | 116.2178 | 31.2561 | 263 | 119.8428 | 27.2561 |
| 44 | 118.5049 | 34.9867 | 154 | 116.3428 | 35.4228 | 264 | 119.8428 | 27.2978 |
| 45 | 100.0511 | 29.1311 | 155 | 116.8011 | 32.1311 | 265 | 119.8845 | 27.0895 |
| 46 | 100.1345 | 26.6728 | 156 | 117.0928 | 36.6311 | 266 | 119.8845 | 27.1311 |
| 47 | 100.2178 | 26.8395 | 157 | 117.1345 | 36.6311 | 267 | 119.8845 | 27.1728 |
| 48 | 101.5928 | 34.9645 | 158 | 117.5511 | 32.8811 | 268 | 119.8845 | 27.2145 |
| 49 | 101.7178 | 32.1311 | 159 | 117.8011 | 30.9645 | 269 | 119.8845 | 27.2561 |
| 50 | 101.8845 | 27.0478 | 160 | 117.8428 | 31.6311 | 270 | 119.8845 | 27.2978 |
| 51 | 101.8845 | 35.2561 | 161 | 117.9678 | 35.2978 | 271 | 119.9261 | 27.2561 |
| 52 | 102.1761 | 31.8811 | 162 | 118.0095 | 32.3395 | 272 | 119.9261 | 27.3395 |
| 53 | 102.1761 | 31.9228 | 163 | 118.0928 | 30.3395 | 273 | 119.9678 | 27.1311 |
| 54 | 102.5095 | 23.7145 | 164 | 118.1761 | 32.2145 | 274 | 119.9678 | 27.6311 |
| 55 | 103.1761 | 31.4645 | 165 | 118.1761 | 32.2561 | 275 | 120.0095 | 26.8811 |
| 56 | 104.0928 | 27.7561 | 166 | 118.2595 | 32.2561 | 276 | 120.0095 | 27.3811 |
| 57 | 104.8011 | 25.5061 | 167 | 118.2595 | 32.2978 | 277 | 120.0095 | 30.6728 |
| 58 | 104.8428 | 26.6311 | 168 | 118.3428 | 29.7145 | 278 | 120.0928 | 26.8811 |
| 59 | 104.8428 | 26.7978 | 169 | 118.3428 | 34.1311 | 279 | 120.0928 | 27.5895 |
| 60 | 105.4678 | 26.1728 | 170 | 118.3428 | 35.0895 | 280 | 120.0928 | 28.6728 |
| 61 | 106.2595 | 26.7561 | 171 | 118.3845 | 31.3395 | 281 | 120.1345 | 30.2561 |
| 62 | 106.3011 | 26.7978 | 172 | 118.3845 | 34.1728 | 282 | 120.1761 | 29.2978 |
| 63 | 106.4678 | 26.6311 | 173 | 118.4261 | 29.5895 | 283 | 120.2178 | 27.3811 |
| 64 | 106.5511 | 26.3395 | 174 | 118.4261 | 31.3811 | 284 | 120.2178 | 27.4228 |
| 65 | 106.5928 | 26.2978 | 175 | 118.5095 | 31.4228 | 285 | 120.2178 | 28.5061 |
| 66 | 106.6761 | 26.4228 | 176 | 118.5095 | 34.7561 | 286 | 120.2595 | 29.2978 |
| 67 | 106.6761 | 26.9228 | 177 | 118.5095 | 35.1728 | 287 | 120.3011 | 31.5895 |
| 68 | 106.6761 | 26.9645 | 178 | 118.5511 | 32.0478 | 288 | 120.3428 | 36.0478 |
| 69 | 106.7595 | 26.6311 | 179 | 118.5511 | 32.0895 | 289 | 120.3845 | 27.5478 |
| 70 | 106.8428 | 29.2561 | 180 | 118.5928 | 32.0895 | 290 | 120.4261 | 29.0478 |
| 71 | 106.9678 | 26.7978 | 181 | 118.5928 | 34.5061 | 291 | 120.4261 | 31.4645 |
| 72 | 106.9678 | 26.8395 | 182 | 118.6345 | 32.2978 | 292 | 120.4678 | 28.9228 |
| 73 | 106.9678 | 33.1311 | 183 | 118.6345 | 34.7978 | 293 | 120.5095 | 29.0478 |
| 74 | 107.3428 | 34.0478 | 184 | 118.6345 | 34.8395 | 294 | 120.5928 | 36.1728 |
| 75 | 107.4261 | 26.5061 | 185 | 118.6345 | 35.0061 | 295 | 120.5928 | 36.2145 |
| 76 | 107.6345 | 34.1728 | 186 | 118.6761 | 31.0061 | 296 | 120.5928 | 36.2561 |
| 77 | 107.7178 | 34.0061 | 187 | 118.6761 | 34.7978 | 297 | 120.6761 | 36.6728 |
| 78 | 107.7595 | 27.0061 | 188 | 118.6761 | 90.9645 | 298 | 120.9678 | 28.2145 |
| 79 | 107.7595 | 28.2145 | 189 | 118.7178 | 34.7978 | 299 | 121.0095 | 29.1728 |
| 80 | 107.8011 | 26.3395 | 190 | 118.7595 | 31.8811 | 300 | 121.1761 | 38.8811 |
| 81 | 107.8011 | 26.6728 | 191 | 118.7595 | 34.7561 | 301 | 121.2595 | 38.8395 |
| 82 | 107.8011 | 27.0061 | 192 | 118.7595 | 34.7978 | 302 | 121.4261 | 30.8395 |
| 83 | 107.8011 | 27.0478 | 193 | 118.7595 | 35.1728 | 303 | 121.4261 | 37.4645 |
| 84 | 107.8428 | 26.7145 | 194 | 118.8011 | 32.0478 | 304 | 121.4261 | 37.5061 |
| 85 | 107.8428 | 26.8811 | 195 | 118.8011 | 32.0895 | 305 | 121.9261 | 29.8395 |
| 86 | 107.8428 | 27.0895 | 196 | 118.8428 | 30.7978 | 306 | 121.9678 | 37.2145 |
| 87 | 107.8845 | 26.8811 | 197 | 118.8428 | 31.9645 | 307 | 122.1761 | 37.3395 |
| 88 | 107.8845 | 27.0895 | 198 | 118.8428 | 32.0478 | 308 | 122.4261 | 37.2561 |
| 89 | 107.8845 | 27.1311 | 199 | 118.8428 | 32.0895 | 309 | 122.7595 | 40.0895 |
| 90 | 107.8845 | 27.2145 | 200 | 118.8845 | 30.8811 | 310 | 122.9261 | 41.0478 |
| 91 | 107.9261 | 26.9228 | 201 | 118.8845 | 31.4228 | 311 | 122.9678 | 39.9645 |
| 92 | 107.9261 | 27.0895 | 202 | 118.8845 | 32.0478 | 312 | 123.3011 | 40.7978 |
| 93 | 107.9261 | 27.1311 | 203 | 118.9261 | 31.0478 | 313 | 123.4261 | 41.7978 |
| 94 | 107.9678 | 26.9645 | 204 | 118.9261 | 31.3395 | 314 | 123.5511 | 41.5895 |
| 95 | 107.9678 | 27.0895 | 205 | 118.9261 | 31.8395 | 315 | 123.5511 | 41.6311 |
| 96 | 107.9678 | 27.1311 | 206 | 118.9261 | 31.9645 | 316 | 123.8845 | 40.7978 |
| 97 | 107.9678 | 27.2145 | 207 | 119.0095 | 31.2145 | 317 | 123.9261 | 40.8811 |
| 98 | 108.0095 | 27.1311 | 208 | 119.0095 | 32.0061 | 318 | 124.0511 | 40.4228 |
| 99 | 108.0095 | 27.2978 | 209 | 119.0511 | 31.2145 | 319 | 124.0511 | 40.4645 |
| 100 | 108.0511 | 27.0895 | 210 | 119.0511 | 32.1311 | 320 | 124.0511 | 40.5061 |
| 101 | 108.0511 | 27.2145 | 211 | 119.0928 | 31.2145 | 321 | 124.0511 | 41.0895 |
| 102 | 108.0511 | 27.2978 | 212 | 119.0928 | 31.7561 | 322 | 124.0928 | 40.1311 |
| 103 | 108.0928 | 27.0478 | 213 | 119.0928 | 32.1311 | 323 | 124.0928 | 40.4228 |
| 104 | 108.1345 | 26.8811 | 214 | 119.1345 | 30.9645 | 324 | 124.0928 | 40.4645 |
| 105 | 108.1345 | 27.0061 | 215 | 119.1345 | 31.5895 | 325 | 124.2178 | 40.0061 |
| 106 | 108.1345 | 28.2561 | 216 | 119.1345 | 31.9228 | 326 | 99.0095 | 28.0061 |
| 107 | 108.1345 | 28.2978 | 217 | 119.1761 | 27.0895 | 327 | 99.3845 | 28.1728 |
| 108 | 108.1761 | 28.3395 | 218 | 119.1761 | 31.9645 | 328 | 99.6345 | 27.7561 |
| 109 | 108.2178 | 26.9228 | 219 | 119.2178 | 29.5895 | 329 | 99.8011 | 27.7978 |
| 110 | 108.2595 | 27.0895 | 220 | 119.2178 | 30.1728 | 330 | 99.8428 | 28.5895 |

Table.S3.Sampling point information of *Pseudostellaria heterophylla*

| NO. | longtitude | latitude | NO. | longtitude | latitude |
| --- | --- | --- | --- | --- | --- |
| 1 | 119.5521 | 27.2567 | 23 | 118.7949 | 30.8637 |
| 2 | 119.5730 | 27.3816 | 24 | 119.2494 | 30.9200 |
| 3 | 119.6698 | 27.1846 | 25 | 118.0884 | 26.9878 |
| 4 | 119.7018 | 27.2248 | 26 | 105.9230 | 26.9885 |
| 5 | 119.7767 | 27.2468 | 27 | 109.1167 | 27.4090 |
| 6 | 119.8198 | 27.2754 | 28 | 108.0220 | 27.2158 |
| 7 | 119.9031 | 27.2032 | 29 | 108.1773 | 27.0689 |
| 8 | 119.9288 | 27.1418 | 30 | 106.2601 | 26.7667 |
| 9 | 119.8598 | 27.1223 | 31 | 107.8784 | 26.1817 |
| 10 | 119.8689 | 27.0502 | 32 | 107.2704 | 25.9278 |
| 11 | 119.9722 | 27.2421 | 33 | 107.9253 | 27.1402 |
| 12 | 120.0046 | 27.1874 | 34 | 106.5864 | 26.2742 |
| 13 | 120.0356 | 27.2565 | 35 | 108.3457 | 27.0058 |
| 14 | 119.3121 | 32.1681 | 36 | 107.4640 | 26.7735 |
| 15 | 119.2863 | 31.7178 | 37 | 108.2400 | 27.0647 |
| 16 | 119.2683 | 31.6809 | 38 | 107.9029 | 27.2128 |
| 17 | 119.1887 | 31.3618 | 39 | 108.2214 | 27.0433 |
| 18 | 116.9111 | 31.3784 | 40 | 107.8918 | 27.1316 |
| 19 | 117.0169 | 31.4515 | 41 | 118.5475 | 34.9143 |
| 20 | 116.3838 | 31.7395 | 42 | 118.3435 | 34.9210 |
| 21 | 119.5363 | 30.8157 | 43 | 118.5727 | 35.3567 |
| 22 | 118.8000 | 30.8052 | 44 | 118.5049 | 34.9867 |

Table.S4. 55 environmental variables

| Abbreviated name | Variable description | Unit |
| --- | --- | --- |
| altitude | altitude | m |
| Aspect | Aspect | ° |
| Slope | Slope | ° |
| zblx | Vegetation type | — |
| index_ci | Cold index | — |
| index_hi | Humidity index | — |
| index_wi | Warmth index | — |
| ntl | Clay content in the upper layer of the soil (0-30cm) | % |
| pH | Soil pH | — |
| YJTHL | Organic carbon content in the upper layer of soil (0-30cm) | % |
| HSL | Sediment content in the upper layer of the soil (0-30cm) | % |
| YXSFHLDJ | Grade of soil available water content | — |
| SoilType | SoilType | — |
| ZDFL | Soil texture classification | — |
| YLZJHN | Cation exchange capacity in the upper layer of soil (0-30cm) | c mol/kg |
| prec1 | average precipitation in January | mm |
| prec10 | average precipitation in October | mm |
| prec11 | average precipitation in November. | mm |
| prec12 | average precipitation in November. | mm |
| prec2 | average precipitation in February | mm |
| prec3 | average precipitation in March. | mm |
| prec4 | average precipitation in April | mm |
| prec5 | average precipitation in May. | mm |
| prec6 | average precipitation in June | mm |
| prec7 | average precipitation in July | mm |
| prec8 | average precipitation in August | mm |
| prec9 | average precipitation in September | mm |
| tmean1 | Average temperature in January | ℃ |
| tmean10 | Average temperature in October | ℃ |
| tmean11 | Average temperature in November. | ℃ |
| tmean12 | Average temperature in December | ℃ |
| tmean2 | Average temperature in February | ℃ |
| tmean3 | Average temperature in March. | ℃ |
| tmean4 | Average temperature in April | ℃ |
| tmean5 | Average temperature in May | ℃ |
| tmean6 | Average temperature in June | ℃ |
| tmean7 | Average temperature in July | ℃ |
| tmean8 | Average temperature in August | ℃ |
| tmean9 | Average temperature in September | ℃ |
| BIO1 | Annual Mean Temperature | ℃ |
| BIO2 | Mean Diurnal Range (Mean of monthly (max temp - min temp)) | ℃ |
| BIO3 | Isothermality (BIO2/BIO7) (* 100) | — |
| BIO4 | Temperature Seasonality (standard deviation *100) | — |
| BIO5 | Max Temperature of Warmest Month | ℃ |
| BIO6 | Min Temperature of Coldest Month | ℃ |
| BIO7 | Temperature Annual Range (BIO5-BIO6) | ℃ |
| BIO8 | Mean Temperature of Wettest Quarter | ℃ |
| BIO9 | Mean Temperature of Driest Quarter | ℃ |
| BIO10 | Mean Temperature of Warmest Quarter | ℃ |
| BIO11 | Mean Temperature of Coldest Quarter | ℃ |
| BIO12 | Annual Precipitation | mm |
| BIO13 | Precipitation of Wettest Month | mm |
| BIO14 | Precipitation of Driest Month | mm |
| BIO15 | Precipitation Seasonality (Coefficient of Variation) | — |
| BIO16 | Precipitation of Wettest Quarter | mm |
| BIO17 | Precipitation of Driest Quarter | mm |
| BIO18 | Precipitation of Warmest Quarter | mm |
| BIO19 | Precipitation of Coldest Quarter | mm |

Table.S5. Using 55 environmental variables to predict the distribution of Pseudostellaria heterophylla in China and the contribution rate of main environmental variables.

| Variables  ID | Description | Contribution (%) | Permutation importance(%) | Units |
| --- | --- | --- | --- | --- |
| prec11 | average precipitation in November. | 42.9 | 8.2 | mm |
| bio18 | Precipitation of Warmest Quarter | 7 | 0.4 | mm |
| bio12 | Annual Precipitation | 5.5 | 0 | mm |
| tmean9 | Average temperature in September | 4 | 25.2 | °C |
| tmean10 | Average temperature in October | 3.5 | 0.2 | °C |
| tmean5 | Average temperature in May | 3.5 | 0.1 | °C |
| prec4 | average precipitation in April | 3.4 | 4 | mm |


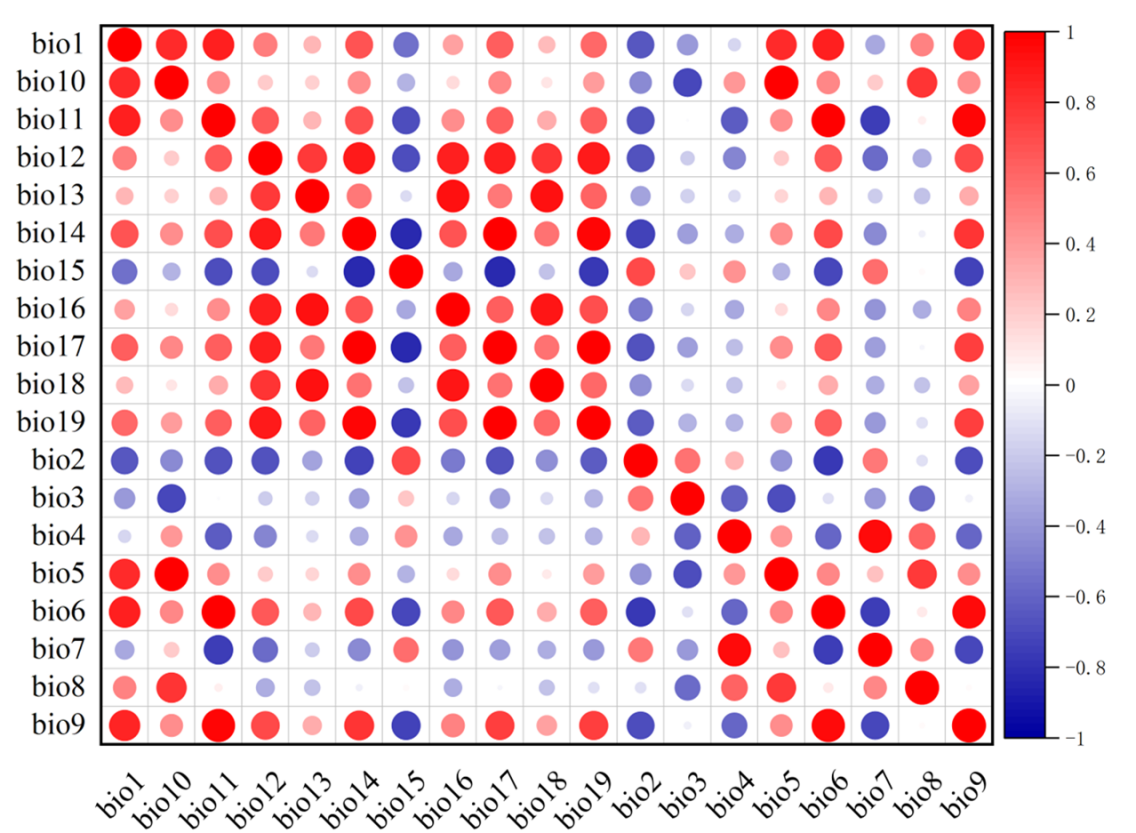


Fig. S1 Correlation coefficient matrix of 19 bioclimate variables.


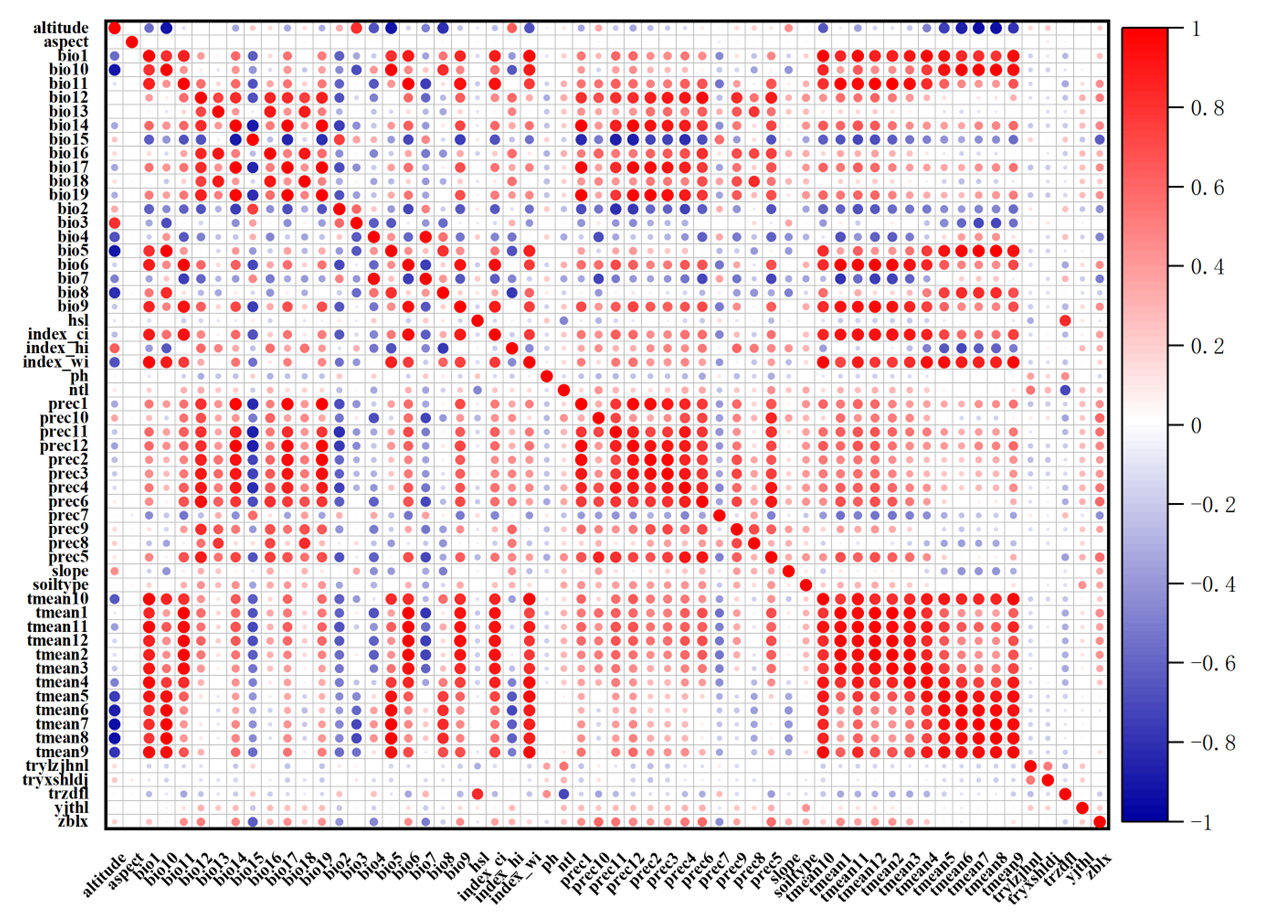


Fig. S2 Correlation coefficient matrix of 55 environmental variables.
